# A Sandwich‐model experiment with personal response systems on epigenetics: insights into learning gain, student engagement and satisfaction

**DOI:** 10.1002/2211-5463.13135

**Published:** 2021-03-29

**Authors:** Georgia Katsioudi, Efterpi Kostareli

**Affiliations:** ^1^ Center for Integrative Genomics University of Lausanne Lausanne Switzerland; ^2^ The Wellcome‐Wolfson Institute for Experimental Medicine School of Medicine, Dentistry and Biomedical Sciences Faculty of Medicine, Health and Life Sciences Queen’s University Belfast Belfast Northern Ireland UK; ^3^ School of Allied Health Sciences Faculty of Health & Life Sciences De Montfort University Leicester UK

**Keywords:** active learning, clickers, higher education didactics, personal response systems, sandwich principle, teaching epigenetics

## Abstract

Current trends in Higher Education Pedagogies include an ongoing discussion about active learning strategies. Technology‐based interventions such as personal response systems (PRS) have gained momentum, especially since the advent of cloud‐/web‐based solutions. One model that supports the transition from traditional lecturing towards active learning by maintaining a balance between instruction and self‐learning is the ‘Sandwich Model’. In the present study, we investigated the impact of the Sandwich Model combined with PRS in student learning, engagement and satisfaction by a randomised trial in a large undergraduate biomedical/medical sciences class. A teaching session on epigenetics was delivered either as a traditional lecture (C‐group) or as a PRS‐including Sandwich‐based session (S‐group). The major finding of our experiment was the significantly enhanced performance of the S‐group over the control, suggesting that the Sandwich Model improves learning gain. We also provide strong evidence that the Sandwich Model enhances student engagement and satisfaction. However, the effect of the Sandwich Model in learning gain and student attitudes was not dependent on PRS incorporation *per se* and students seemed to favour non‐PRS activities over PRS, as evidenced by their feedback. Although further experimental research is needed in order to conclusively compare and contrast PRS and non‐PRS activities regarding learning gain, we propose the usage of the Sandwich Model with a variety of in‐class learning activities, both PRS and non‐PRS‐based. Altogether, our work shows that the Sandwich Model is a powerful pedagogical approach that exerts a positive impact on student perceptions for learning and satisfaction and that can support the teaching of challenging biomedical concepts, such as epigenetics.

## Graphical abstract

Here, we investigate the effect of the didactic model termed ‘the Sandwich model’ on teaching epigenetics in a randomised trial of biomedical undergraduates. A variety of active learning activities, both PRS*‐based and non‐PRS, were incorporated within the Sandwich Model. Study results provide significant evidence for important educational benefits upon Sandwich application regarding in‐class learning, student engagement and satisfaction (*PRS = personal response systems).

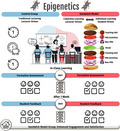

**Abbreviations**
C‐group: control group of students
ICAP: Interactive, Constructive, Active, and Passive (The ICAP Framework and hypothesis)
PRS: personal response systems
QUE1: questionnaire 1 (MCQ testing learning in‐class)
QUE2: questionnaire 2 (Feedback)
S‐group: Sandwich model group of students

Political, economic and social factors over the past decade underlie the massification of higher education [1]. The pressure for higher student intake is transforming the educational process, with important consequences for the quality of teaching delivery. Large course enrolment is a current trend in medical and biomedical courses in the UK and other countries and is regarded a pure challenge for the academic lecturer [2, 3]. In parallel with the increase in student intake, there has been an ongoing discussion about the active learning definitions and benefits [4]. A significant number of research studies have demonstrated that active learning strategies have important educational benefits [5, 6, 7, 8, 9, 10, 11]; however, traditional lecturing is reported to be still the norm for large classes [2, 12, 13]. Among the identified reasons for keeping up the passive forms of teaching are limitations on lecturers' time resources and institutional support, as well as concerns about teaching evaluation [14, 15, 16]. Interestingly, a recent pioneer study [17] reported an inherent student bias against active learning, which may have implications for active learning benefits. This bias can be reflected in reports of student satisfaction and perception of learning in‐class.

Among active learning approaches, one solution that has been suggested to improve student experience is the ‘Sandwich Model’ or ‘Sandwich Principle’ [18, 19]. The Sandwich Model is a didactic approach, which places emphasis on individual learning in‐class. This teaching concept is enabled by a characteristic ‘architecture’ with periodical alternation between collective learning (lecturer‐driven) phases and individual (active learning, student‐driven) phases. Lectures are broken down into smaller deliverables often in junction with learning objectives. A rather strict structure and flow of the teaching session seems to be a core feature of this model. The interlude between two learning packages can be any active learning strategy such as a quiz, small group discussion and problem‐based learning activity. The Sandwich Model architecture is depicted in the literature as a sandwich or burger with bread to represent introduction and conclusion, patties the main body phases of collective learning, whereas vegetables or cheese slices are used for the phases of individual learning (motivation, processing, memorisation and transfer of knowledge and skills) (Fig. 1B,C). The collective learning phases are more compact and often demanding, whereas the self‐learning phases may follow a graduated difficulty. The Sandwich Model development was based on the consideration of attention span [20, 21, 22]. Taking into account the scepticism about attention span studies [22] in addition to the lack of randomised trial data, a systematic evaluation of the model’s impact on teaching outcome is currently needed.

**Fig. 1:**
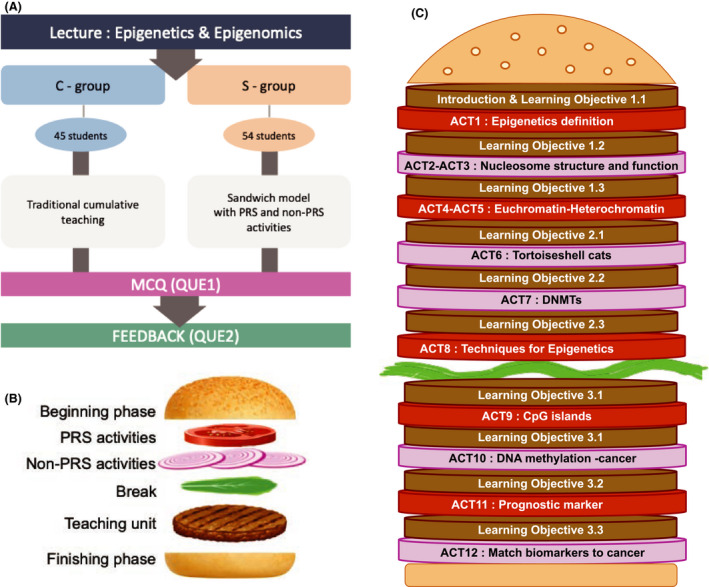
Study design and the architecture of the Sandwich model. (A) Summary of study design. In total, 99 students participated in the study. Of them, 45 were taught by the traditional way of lecturing (C‐group) and 54 by the sandwich model including PRS activities (S‐group). In‐class learning was evaluated at the end of each teaching session with an identical for both group questionnaire (QUE1). (Appendix S2). A week later, students were asked to provide feedback on their experience and learning by a second questionnaire (QUE2, Appendix S3). (B) Sandwich model with PRS. Schematic representation of the sandwich model. The beginning and finishing phases are depicted as the burger bun. PRS activities are presented by a slice of tomato and the non‐PRS activities by sliced of onion. The burger stake represents the main teaching units delivered by the lecturer (collective learning). The break in the middle of the lecture is depicted as a piece of lettuce. (C) Diagram of the sandwich architecture of our study experiment. PRS activities are represented in red (tomatoes) and non‐PRS in light magenta (onions). Details on learning objectives (brown, meat) can be found at the presentation source file (Appendix S1). The activities are placed in chronological order.

The Sandwich Model can be viewed as a conservative approach that keeps a balance between traditional lecture elements (i.e. burger stakes, lecturer deliver key concepts) and active learning activities (tomatoes/onions) (Fig. 1B,C). Active learning sessions can be based on a wide range of learning techniques such as experiential learning, learning by doing, gamification, peer tutoring and peer discussion, the muddiest point, one‐minute paper, quizzes and many more techniques which can often involve technology such as the use of personal response systems (PRS) [23]. Most higher‐educational institutions nowadays offer PRS solutions with the use of either clickers or mobile devices. A significant number of studies [23, 24, 25, 26, 27, 28, 29] suggest that PRS could be an efficient solution for incorporation of active learning practices. Although PRS can increase student engagement in class, one should not ignore work indicating that incorporation of PRS may have little effect on actual learning and students’ performance on assessments [30].

In the present study, we performed a randomised trial (Fig. 1) on the challenging topic of epigenetics in a large undergraduate class of medical and biomedical sciences in order to obtain systematic evidence for the effect of the Sandwich Model with incorporation of PRS on (a) enhancing in‐class learning, (b) formative assessment performance and (c) student perceptions related to engagement and satisfaction. Our starting point and working hypothesis were that lectures structured with the Sandwich Model support active learning, enhance student engagement and learning experience and that PRS is an easy to integrate active learning strategy within the Sandwich architecture applicable for large classes. Our experimental data confirm this hypothesis and provide valuable insights into the value of PRS and non‐PRS active learning strategies.

## Materials and methods

### Sampling

Study participants were undergraduates of ‘Biomedical Science BSc (Hons)’ and ‘Medical Sciences BSc (Hons)’ at De Montfort University, Faculty of Health and Life Sciences, UK. The experimental setting included randomised splitting of the class for the module BIOM2001‐‘Molecular Genetics and Genomics’ (Level 5) into two groups (126 vs. 115) by an online randomiser platform (https://www.random.org/lists/) and delivering the epigenetic lecture with applying the Sandwich Model (S‐group) or by traditional lecturing (control: C‐group), respectively. Recruitment strategy was based on (a) an in‐class announcement 2 weeks before the experiment by the lecturer/module leader as well as announcements at blackboard (EK); (b) action of year representatives for advertising the project among their peers and (c) a raffle for participants including gifts for three participants.

### Experimental setting

A lecture was delivered by a single instructor (EK) in the two groups of students in consecutive sessions. The experiment was performed once, so a single 2‐h lecture was delivered for each group. The learning objectives were identical for both groups as well as the slides related to the major content (Appendix S1). The control group (C‐group) attended an instructor‐centred lecture (traditional lecturing) on epigenetics and epigenomics. Students were exposed to a rather passive form of learning for two didactic hours (ca. 50 min) with one break in between (Fig. 1A). The S‐group attended the same lecture, but with Sandwich Model architecture and PRS (Turning Point by Turning Technologies, UK). Students of the S‐group spent considerable time on active learning activities (ca. 15 min) facilitated by the instructor. This time difference resulted in a longer break for C‐group and a slightly earlier closure (ca. 7‐min difference for each didactic hour) (Fig. 3A). The presentation for the S‐group was modified as to incorporate principles of the Sandwich Model (Appendix S1). At the end of each session, an identical questionnaire (QUE1, Appendix S2) was employed for assessing student learning in‐class (hard copy/fully anonymised). For the S‐group, each question was linked to a planned active learning activity (the so‐called joints, depicted as ‘tomatoes’ or ‘onions’ at the sandwich architecture [18, 19] (Fig. 1B). Altogether, 12 activities were planned (Table 2, Table S3, Fig. 1B). For 6/12 activities, PRS were used. For non‐PRS interludes, activities such as think–pair–discuss, drawing and explaining a concept, one‐minute paper and peer teaching were employed (Appendix S1, Table 2). On a feedback session 1 week later, students were given a questionnaire (QUE2, Appendix S3) in order to evaluate their experience with the lecturer and the subject. The QUE2 was comprised of nine questions identical for both groups on a five‐point scale (strongly disagree to strongly agree, F1–F9) and six additional questions for the S‐group (F10–F15) as well as three text questions for comments for both groups (F16–F18). In order to check whether our randomised study design had provided us with two groups of comparable or equal prior knowledge and skills, we retrospectively employed as indicator students’ first year overall results (Fig. S2).

### Ethical considerations

The current study was approved by the Research Ethics Committee of the Faculty of Health & Life Sciences at De Montfort University, Leicester, United Kingdom (Ref: 1889). Ethical guidelines as prescribed by The British Educational Research had been followed [31]. The lecture was an inherent part of the module and thereby attendance was obligatory. Students knew their timetabled session well in advanced. The participation in the study was optional; therefore, there was no obligation to complete neither QUE1 nor QUE2. Participants were given a detailed information sheet as well as a consent form to fill in. An information sheet was circulated the week before the experiment on the module sessions. Both C‐group and S‐group teaching sessions were recorded by Panopto Software, and students had access for 48 h to recordings after the feedback session. Furthermore, we offered an optional repetitive session 1 week afterwards for anyone who did not manage to attend or any C‐group member who would like to have an interactive session. This was clearly communicated at the lecture and via blackboard and live at lecture hall.

### Result analysis

The average scores for learning in‐class questionnaire (QUE1) per student/per group were calculated. Comparisons for the six active learning activities within S‐group for PRS and non‐PRS activities were performed by paired *t*‐test to evaluate significance. A repeated‐measure ANOVA was run to examine the effects of the Sandwich Model application and PRS vs. non‐PRS activities. Feedback questionnaire (QUE2) results for the five‐point scale (strongly disagree to strongly agree) were compared among groups with Mann–Whitney *U*‐test for questions F1–F9. For S‐group‐only questions (F10–F15), descriptive statistics were assessed (Table S6). The free‐text question comments (F16–F18) were manually processed by three independent blinded observers and assigned to a five‐point scale (very negative–negative–neutral–positive–very positive) (Table S7). The median of the three evaluators was used for further comparisons and data visualisation (Mann–Whitney *U*‐test). Student engagement and student satisfaction were explored on this basis in order to examine the effect of the Sandwich Model and incorporation of PRS in these key areas. A Likert graph (R package) for the C‐group vs. the S‐group was created, and Mann–Whitney *U*‐test was performed. *P*‐value was considered significant (*P < 0.05), very significant (**P < 0.01) or highly significant (***P < 0.001). For generating plots and statistical analyses, graphpad prism 8.0 (GraphPad Software, La Jolla, CA, USA), stata (STATA 16 software, StataCorp LLC., College Station, TX, USA) IC16.1 and r (R Core Team, Auckland, New Zealand) were utilised.

## Results

### Does the Sandwich Model enhance learning in‐class?

**Fig. 2:**
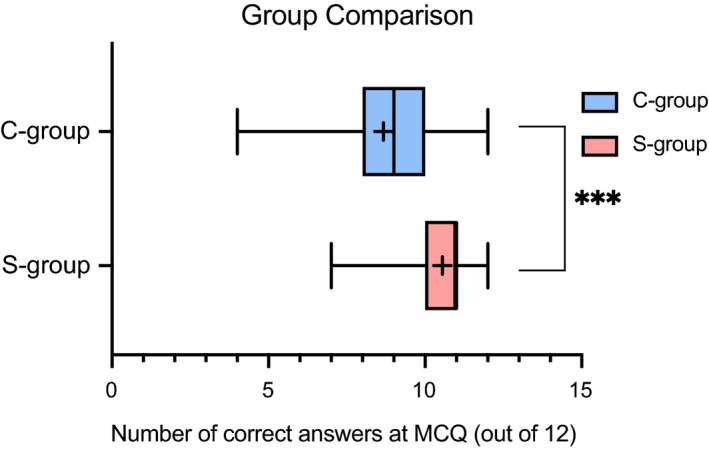
Comparison of performance at MCQ test (QUE1) between the control group (C‐group, traditional lecture, *n* = 45) and the hypothesis testing group (S‐group, sandwich model, *n* = 54) (unpaired *t*‐test: *P* < 0.001, two‐tailed, *t* = 5.861, df = 97).

The student cohort was divided randomly (126 vs. 115) into two groups (morning: C‐group and afternoon: S‐group) (Table 1). The attendance patterns were similar for both sessions and corresponded to the average module attendance throughout the year. Overall, 45 students from the morning session agreed to participate in the study (C‐group) and 54 from the afternoon session (S‐group) (Table 1). When we compared the performance of C‐group vs. S‐group on MCQ test (QUE1, Tables S1,S2), we found that the S‐group scored significantly higher on the QUE1 (*M* = 10.56, SD = 1.19) than the C‐group (*M* = 8.67, SD = 1.97); *t*(97) = −5.86, *P* < 0.001, unpaired *t*‐test) (Fig. 2), suggesting that the S‐group could be advantaged by the Sandwich Model. To further support the link between the higher performance of the S‐group and the application of the Sandwich Model, we verified that the two groups had comparable prior knowledge and skills by evaluating the results of the first academic year as an indicator (Fig. S2). No significant difference was observed between the two groups in terms of prior demonstrated academic skills as first‐year (Level 4) students.

**Table 1:** Student cohort, attendance and participation in the present study. MCQ refers to a formative assessment given at the end of the class to study participants of both C‐group and S‐group (QUE1, Appendix S2) in order to comparatively assess learning in‐class.

	C‐group	S‐group	Total
Enrolled in BIOM2001 module	126	115	241
Present	66	68	134
Absent	60	47	107
Attendance (%)	52.38%	59.13%	55.60%
Study participants	45	54	99
MCQ Participation/Attendance (%)	68.18%	79.41%	73.88%

### Does the Sandwich Model support an extended attention span?

**Fig. 3:**
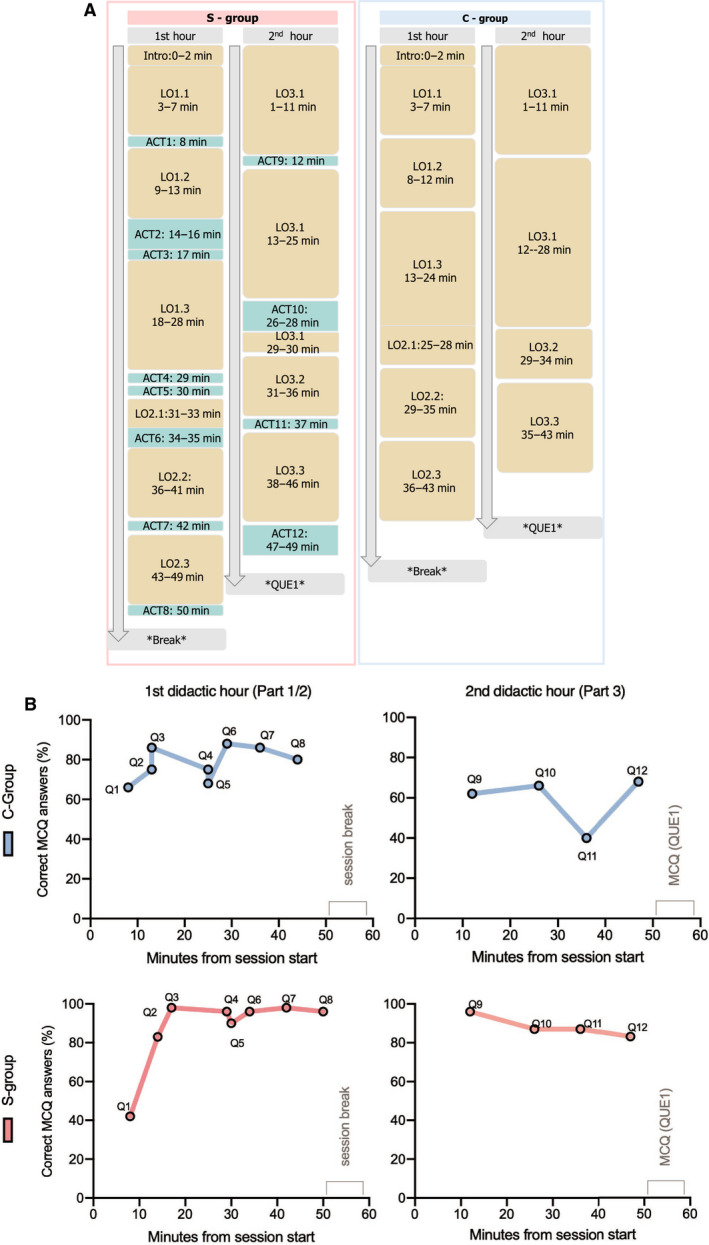
The Sandwich Model and attention span: (A) Schematic representation of delivery design with learning objectives (LO) and active learning activities (ACT) for the S‐group and C‐group, respectively. Details on learning objectives and activities can be seen at Appendix S1 and Table S3. (B) Time slot of content delivery and learning outcome for the C‐group and S‐group. Corresponding questions (Q1…Q12) from the QUE1 (Table 2, Table S3) are associated with the time point of delivery within didactic hours. *Y*‐axis shows the percentage of correct answers per each question. *X*‐axis shows the time slot (minutes from lecture start) rounding the minute (i.e. slide related to Q1 was presented at 7’35’’ taught). It is important to underscore that S‐group was offered 12 interactive activities of a total duration ca. 15 min. In reality, the C‐group had a rather longer break and a slightly earlier lecture closure (ca. 8 min).

Learning activities at the S‐group session were spread across the 2‐h lecture as to have a regular interchange between phases of collective learning (burger stakes; Figs 1B and 3A) and phases of self‐learning activities, either at the form of a PRS question (tomatoes; Figs 1B and 3A) or at the form of a non‐PRS activity (onions; Figs 1B and 3A). For the C‐group, two long collective learning phases (Fig. 3A) were delivered. A detailed scheme on time management and content delivery for the two sessions is depicted in Fig. 3A. Interestingly, when we compared the results per question in association with the time slot of delivery, we observed that with exception of Q1, the S‐group had a rather stable learning in‐class overtime (Fig. 3B). As far as the C‐group is concerned, we observed that attention or ability to learn in‐class had two important lows at 35 min of the first didactic hour and at 35 min of the second didactic hour. Overall, the S‐group performed in 11/12 questions better than the C‐group and the difference was higher at the second didactic hour (Fig. 3B). A small degree of decrease in the ability to learn in‐class was observed also for S‐group at the second didactic hour (Fig. 3B). However, it is important to underscore that because of the incorporated activities at the S‐group session, there was a 14‐ to 15‐min difference between S‐group and C‐group in teaching duration as shown at Fig. 3A.

### Do personal response systems within the Sandwich Model support learning in‐class?

**Fig. 4:**
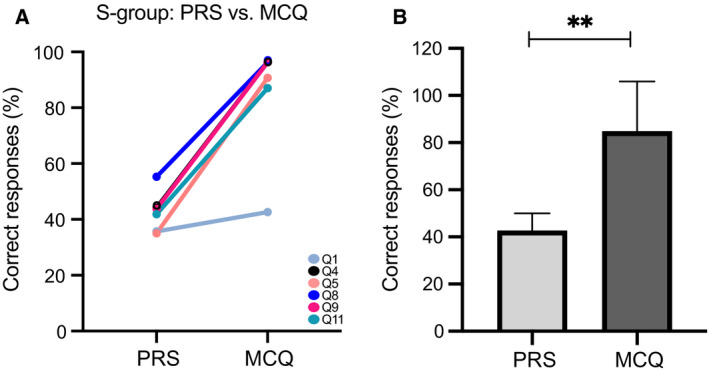
Comparative results for PRS and MCQ for S‐group. Six questions were presented at the S‐group (*n* = 54) as multiple‐choice questions with Turning Point in‐class at various time points across the 2 h of lecture, and they were part of the MCQ test (QUE1) at the end of the teaching session (see also Table S4). (A) The correct responses as percentage per question are depicted for PRS and non‐PRS (MCQ) setting. A clear increase on 5/6 questions was observed. (B) The score of correct responses was significantly improved at the MCQ in comparison with PRS setting (mean with SD is depicted) (**: paired *t*‐test; *P*‐value = 0.0023, two‐tailed, *t* = 5.702, df = 5, SD = 18.15, SEM = 7.408, 95% CI = 23.20–61.28, *R* ^2^ = 0.867).

We employed PRS on six occasions (Table 2) within the Sandwich Model session (S‐group). Student engagement with PRS technology was 74–89% (Table S4) per activity/question. Results from the PRS activities are depicted at Fig. S1. For 5/6 questions, the majority of the class spotted the right answer already from the PRS activity (not disclosed prior to QUE1 assessment). When we comparatively accessed the performance to the same six questions at the PRS vs. the formative assessment (QUE1, Appendix S2), a highly significant increase in correct responses was observed (Fig. 4B). At single question level, the improvement of scores was impressive for Q4, Q5, Q8, Q9 and Q11 (Fig. 4A). The Q1 was the only question in which the S‐group scored in general less as compared to the C‐group (Fig. 3B).

**Table 2:** List of integrated active learning activities within the lecture and corresponding question code at QUE1. Details for each question/activity can be found at Appendix S1, S2.

	Question	Activity	Activity/Question theme	PRS	Time activity occurred (min)
1st hour	Q1	ACT1	Epigenetic definition	+	8
	Q2	ACT2	Nucleosome structure	−	14–16
	Q3	ACT3	Nucleosome function	−	17
	Q4	ACT4	Euchromatin	+	29
	Q5	ACT5	Heterochromatin	+	30
	Q6	ACT6	Tortoiseshell cats	−	34–35
	Q7	ACT7	DNMTs	−	42
	Q8	ACT8	Techniques for epigenetics	+	50
Break (10‐min)					
2nd hour	Q9	ACT9	CpG islands	+	12
	Q10	ACT10	DNA methylation – cancer	−	26–28
	Q11	ACT11	Prognostic marker	+	36
	Q12	ACT12	Match biomarkers to cancer	−	47–49

**Fig. 5:**
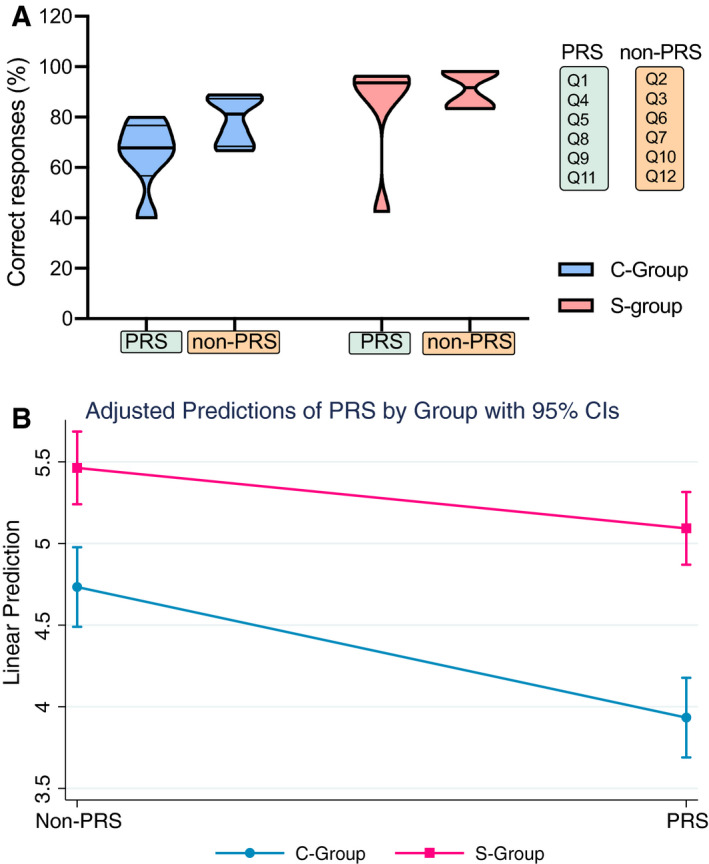
(A). Correct responses per group divided by category of questions (PRS‐employed or non‐PRS activities). For C‐control group, no activities were involved but the scores on the same MCQ questions (highlighted in light orange for non‐PRS and green for PRS) were employed. (B) Adjusted predictions of PRS question categories by group with 95% CIs.

However, from an educational point of view it is unclear whether PRS‐based learning activities within the Sandwich Model are superior to other active learning activities employed (non‐PRS) (Table 2 and Table S3). In order to get insights into this issue, we compared results from QUE1 linked to learning activities for the S‐group (Fig. 5) and with that of the C‐group that answered the same questions without any intervention. By a repeated‐measures ANOVA, we examined the effects of the Sandwich Model application including PRS/non‐PRS activities in students’ performance (Table 3). We observed a statistically significant main effect for group comparison [*F*(1, 97) = 34.35, *P* < 0.001, partial η^2^ = 0.26], which indicates that the average score across the non‐PRS and PRS questions differed significantly between the two groups (Fig. 5A). The S‐group (M_Non‐PRS_ = 5.46, SD_Non‐PRS_ = 0.75; M_PRS_ = 5.09, SD_PRS_ = 0.78) outperformed the C‐group (M_Non‐PRS_ = 4.73, SD_Non‐PRS_ = 1.16; M_PRS_ = 3.93, SD_PRS_ = 1.25) based on descriptive statistics. Interestingly, there was also a statistically significant main effect for PRS [*F*(1, 97) = 24.75, *P* < 0.001, partial η^2^ = 0.20]. This indicates that on average, the students’ scores differed between the non‐PRS and PRS questions at the C‐group, with students to score higher on non‐PRS questions. Hypothesis testing for the C‐group confirmed that there is a significant difference between the QUE1 results for PRS and non‐PRS questions and a nondistribution among the two categories (Fig. S3). These results point towards a potential ‘inherited’ imbalanced at difficulty level between PRS and non‐PRS questions. Finally, an examination of interaction effects revealed that there is no statistically significant interaction between group and scores at PRS questions [*F*(1,97) = 3.33, *P* = 0.07, partial η^2^ = 0.03], implying that the degree of effect of PRS is not dependent on the group (Fig. 5B).

**Table 3:** Results of repeated‐measures ANOVA in order to examine the effects of the Sandwich Model application and personal response activities (PRS). *N* = 99 students/Total observations = 198.

Source	SS	df	MS	*F*	*P*
Between‐subjects					
Group (C vs. S)	185.56	100	1.86	2.73	< 0.001
Error	123.67	97	1.27		
Within‐subjects					
PRS	16.81	1	16.81	24.75	< 0.001
PRS × Group	2.27	1	2.27	3.33	0.07
Error	65.90	97	0.68		
Total	251.45				

### How student perceive the Sandwich Model, PRS and other active learning techniques?

**Fig. 6:**
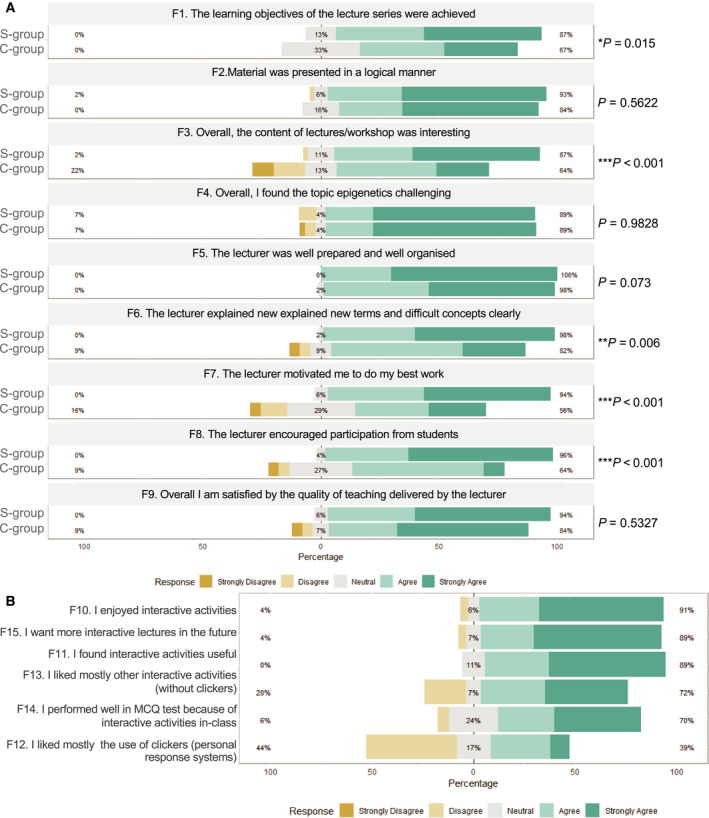
Students’ Feedback and Perceptions for the Sandwich Model and PRS: Results from QUE2 depicted by Likert R package. (A). A comparative view between C‐group and S‐group is provided per question for the F1–F9 questions which both groups answered. (*P*‐values, Mann–Whitney *U*‐test) (B). Results for the Sandwich‐related questions (F10–F15) which were asked only to S‐group cohort (see also Appendix S3, QUE2 and Tables S5,S6).

In order to examine student engagement and satisfaction linked to the Sandwich Model application with PRS and non‐PRS activities, we provided a feedback questionnaire (QUE2, Appendix S3) to both experimental conditions 1 week after the experiment. The first part of the feedback questionnaire was comprised by nine questions (F1–F9) using a 5‐point Likert scale (from strongly disagree to strongly agree). For the S‐group, six additional questions related to active learning strategies had been included (F10–F15) (Fig. 6, Table S5). In general, the whole‐study cohort demonstrated overall positive attitudes towards the lecturer and the topic (Fig. S4). When we comparatively assessed feedback, we observed that the S‐group gave consistently higher feedback scores across all questions (Fig. 6A). For instance, the S‐group responded that ‘the learning objectives of the lecture series were achieved’ (F1) significantly higher than the C‐group (*P* = 0.015, Mann–Whitney *U*‐test) and rated that the ‘content of the lectures was interesting’ (F3) much more highly than the C‐group (*P* < 0.001) (Fig. 6A). Regarding lecturer’s ability to ‘explain new terms and difficult concepts clearly’ (F6), the S‐group outrated the C‐group (*P* = 0.006). Also, the S‐group reported higher motivation levels (F7, *P* < 0.001). Finally, the S‐group reported that the lecturer encouraged participation more than the C‐group (*P* < 0.001). Regarding questions F10–F15 (S‐group only), descriptive statistics were calculated (Table S6) and a corresponding Likert was created (Fig. 6B). The highest ratings were achieved for questions F10 – ‘I enjoyed interactive activities’ (91% strongly agree), F15 – ‘I want more interactive lectures in the future’ and F14 – ‘I performed well in the MCQ test because of interactive activities in class’. The lowest rating was for F12 – ‘I mostly liked the use of clickers (PRS)*’* (39% strongly agree) (Fig. 6B).

**Fig. 7:**
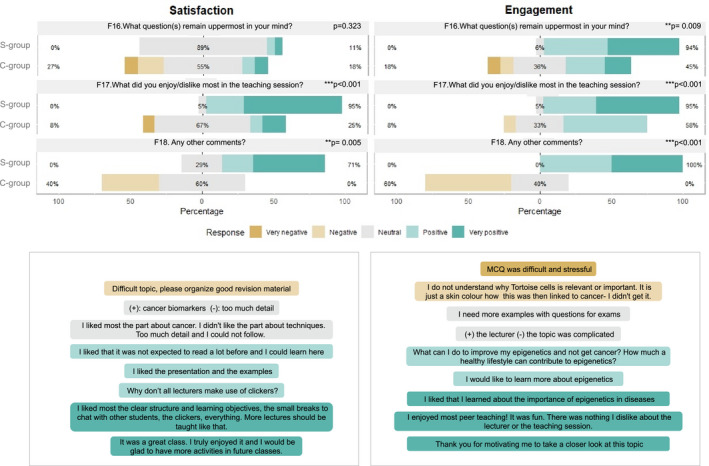
Analysis of students’ free‐text feedback regarding student engagement and satisfaction: Likert charts arising from the three free‐text feedback questions (QUE2, F16–F18) related to student satisfaction (left up) and engagement (right up) for the S‐group and the C‐group. Evaluation of comments was performed by three independent observers who scored comments in a five‐point scale (from very negative to very positive). The median score of the three evaluators was used to classify each comment to one of the five points (Table S7). *P*‐values were calculated by Mann–Whitney *U*‐test. Sample comments for each of the coding category are provided at the lower panels for satisfaction and engagement, respectively. A full list of all comments received per group of study and their assigned coding category can be found at Table S7.

Three free‐text commenting opportunities were included in the second part of QUE2 (Appendix S3) (F16–F18). We manually processed all the free‐text comments, and we assigned them to a five‐point Likert scale (very negative to very positive) (Table S7) both for student engagement and for satisfaction. The median of three independent evaluators was used. As shown in Fig. 7, C‐group comments for student engagement are approximately normally distributed around neutral (36%) and positive comments (36%), and around neutral comments (60%) for student satisfaction, with 21% negative/very negative comments in both cases. The S‐group did not have any negative/very negative comments; on the contrary, 53% were very positive and 43% were positive for student engagement, and 41% were neutral or positive for student satisfaction. C‐group participants were more concerned with whether the material learnt was relevant for examinations and obtaining more examples and questions to help them in that regard. S‐group participants were significantly more engaged; over 90% of the comments were positive/very positive. They asked for more details about biomarkers and their relation to cancer and also enjoyed the interactive nature of the lesson. In terms of student satisfaction, C‐group participants were comparatively overwhelmed with the level of detail and pace and they considered the topic to be difficult. S‐group participants, on the other hand, enjoyed the active learning strategies incorporated into the lessons and would like them to be incorporated into other lectures as well (F17 and F18). They enjoyed both the interactive activities, including the use of clickers as well as the discussions with other students.

To determine the statistical significance of the difference between the C‐group and S‐group, regarding student engagement and satisfaction, we performed Mann–Whitney U‐test. The category of comments on the five‐point scale for student engagement/satisfaction stratified by group revealed significant difference for 4/6 comparisons (Fig. 7). Overall, we found that the increased frequency of positive comments in the S‐group, compared with the C‐group, is statistically significant when participants were asked what they liked/disliked in the sessions (F17). The considerable increase in positive ‘additional comments’ made by S‐group participants, in comparison with the C‐group, is highly significant for engagement and satisfaction (F18, *P* < 0.001 and *P* = 0.005).

## Discussion

The concept of epigenetics has emerged as a highly important branch of genetics with remarkable links to human health [32, 33]. Teaching epigenetics on large cohorts can be a real challenge for the academic lecturer, in particular when the students have no solid foundation on molecular biology and/or genetics. Therefore, we consider this topic as a great opportunity to perform a Sandwich‐based educational experiment and measure both student learning in‐class and student perceptions. In order to get insights for the effectiveness of the Sandwich Model [18, 19, 34] with incorporation of PRS, we designed an ‘in‐class’ Sandwich‐based experiment during which identical content was delivered in randomised student populations.

### The Sandwich Model enhances learning in‐class

Firstly, we aimed at assessing the broad applicability of the Sandwich Model‐based (S‐group) teaching delivery. Here, we report a highly significant enhancement of learning in‐class and formative assessment performance upon the Sandwich Model application. Although this is the first large randomised trial ever performed under a time bound and strict designed delivery frame, further work in larger cohort and in various STEM disciplines, with more than one experimental repeat, would have been valuable for drawing firm conclusions. Furthermore, a comparative analysis between the Sandwich Model which can be viewed as a rather conservative active learning strategy and a more progressive model with limited or no collective learning phases such as the flipped‐classroom model [35, 36] would have provided key pedagogical insights into the ‘right‐dose’ and the ‘right‐type’ of active learning activities and the underlying mechanisms for the positive outcomes and learning gain related to the Sandwich Model application. This is of particular importance considering that meta‐analyses of active learning pedagogies such as the Flipping emphasise the need to consider several parameters upon application, in order to address the efficiency of a pedagogical strategy. For instance, flipping the classroom was efficient when the face‐to‐face contact was not reduced and when quizzes were incorporated on the design [37]. In an analogy to this notion, one could argue that the Sandwich Model seems to be efficient when one incorporates a good mix of different activities. This might include quizzes with the aid of PRS as evident by formative assessment results on our experiment, as well as student feedback.

An important question that emerges from the aforementioned line of argumentation is whether the observed superior results of the S‐group are causally linked to the application of the Sandwich Model. Despite the fact that our study provides strong evidence for a positive role of the Sandwich Model in enhancing students’ learning in‐class, the nature of the MCQ (i.e. repetition of questions) and various confounding variables should be discussed and investigated in follow‐up studies before attributing any causality to a single teaching model. For instance, it could be argued that there was an overlap between QUE1 questions and PRS questions in the S‐group session, and therefore, improved assessment results may demonstrate that students just perform better due to repetition (‘two attempts’ in the same question). Although this is a reasonable scenario for 6/12 questions, it is important to note that for the 6 non‐PRS‐related questions, the S‐group students performed even better. If the ‘two‐attempt scenario’ was true beyond and above all other parameters, one could have expected that the S‐group would have performed significantly better at the PRS‐related questions in comparison with non‐PRS. Furthermore, when we compared the C‐group vs. the S‐group for the six non‐PRS 6/12 questions (Q2, Q3, Q6, Q7, Q10) (Table S3), we found that still S‐group performs significantly better (*P* = 0.0349, unpaired *t*‐test). However, for a systematic investigation of the learning gain related to the Sandwich Model, different approaches in regard to formative assessment and large comparative trials evaluating the Sandwich Model vs. nonhighly structured teaching models are required.

Another important aspect is the impact of potential confounding variables, such as prior student knowledge and skills or the time slot that the lecture was delivered. Interestingly, when we compared first‐year results between the S‐group vs. the C‐group, we observed that overall the final mark distribution was balanced between the two groups (Fig. S2). Therefore, we argue that randomisation has worked well for this cohort and that the S‐group was not academically significantly more talented than the C‐group. This excludes potential imbalanced prior knowledge as a significant parameter, based of course on the assumption that the first‐year overall mark is a good indicator to compare prior knowledge and performance potential.

As far as the time slot of lecture delivery (morning: 09.00–11.00 vs. afternoon 13.00–15.00) and how this could have influenced our trial is concerned, existing literature provides contradictory evidence. On the one hand, studies since decades have reported that students tend to perform better in the morning [38], whereas on the other hand studies report higher absenteeism and low student engagement in early morning lectures linked to certain chronotypes [39]. Based on the general idea that students performed better in the morning, one would have expected the C‐group (morning) to achieve higher scores than the S‐group (afternoon), which is at variance with our results. In fact, the S‐group performed better despite an afternoon‐slot delivery. Of course, a nonbalanced distribution of different chronotypes among study groups could theoretically have impacted on our study results [39, 40]. Therefore, further studies are required in order to shed light into potential co‐factors such as the one discussed above and to check causality between performance and the Sandwich Model. Our study provides a strong rational for further future research on this area and suggests that the Sandwich Model can be seen as a powerful didactic model which worth more attention.

### The Sandwich Model and attention span

A rather simple explanation about the efficiency of the Sandwich Model in the literature is the link between instruction and attention span [18, 19, 21, 41]. At our experiment, learning activities were spread across the 2‐h lecture in an attempt to consider the ‘attention span’ concept (Fig. 3A). Interestingly, our analysis (Fig. 3B) indicates a rather stable learning in‐class overtime for the S‐group in line with the attention spam theory. However, it is difficult to define the exact length of the optimal intervals for attention just by this experiment. The two observed low points at 35 min at first and second didactic hour are not actually directly measuring the attention of students. Therefore, although it seems rational to discuss about attention span, students enhanced engagement and in‐class learning could be also explained by other theories, such as the ICAP hypothesis [42]. Furthermore, a word of caution is needed concerning the time difference between the S‐group vs. the C‐group sessions due to the duration of the interactive activities. With the 12 interactive activities at the S‐group session, one could not claim that the 2‐h sessions were identical (Fig. 3A) and directly comparable in terms of attention span. Overall, there is a clear difference of 14–15 min. The C‐group had therefore a longer break between first didactic hour and second didactic hour and a slightly earlier closure (7 min) than the S‐group. One could argue that the longer break or the earlier completion affects attention span of the C‐group. In this context, our data can serve only as an indication for a potential trend, but not as a strong proof of attention span‐related explanations for the effect of the Sandwich Model.

### PRS within Sandwich Model and learning outcome

Technology‐assisted teaching solutions have been gaining momentum over the past decade. One solution with already extensive presence in higher education is the PRS. PRS are referred to in literature also as Audience Response Systems or In‐Class Voting technologies and can be clicker‐based, web‐based or mixed. Examples of PRS solutions used in academia are the Turning Point, Socrative, eduVote, Poll Everywhere, Communicubes and Mentimeter solutions [28, 43, 44, 45, 46, 47, 48, 49, 50]. Numerous reports discuss their application in Medical and Biomedical Education. However, although most studies report positive student attitudes towards PRS, the data for the effect of PRS on learning outcome and assessment performance are inconsistent [26, 51, 52].

Our own experimental data provided some evidence for a positive correlation between PRS application and enhanced learning in‐class. As seen in Fig. 4 for the PRS questions, students of the S‐group demonstrated improved scores at corresponding formative assessment questions (PRS‐related questions at QUE1) in comparison with the PRS activity scores (see also Table S4). However, a firm conclusion cannot be drawn, since there was an overlap in questions between PRS activities and QUE1 and the improved scores may be due to repetition. It is important to underscore though that students were not exposed to the correct answers during the experiment, but only afterwards.

Next, we asked the question whether PRS activities are superior to non‐PRS activities. Our analysis showed that on average student score differed between the non‐PRS and PRS‐related questions and based on descriptive statistics students scored higher on the non‐PRS questions. By examining the interaction effects between group (S‐group vs. C‐group) category and PRS, we found that the degree of effect of PRS is not dependent on the group/intervention (Clickers Application) (Fig. 5B, Table 3). This is also reflected on the fact that the difference between PRS and non‐PRS scores is also found at the C‐group, in the absence of any intervention. This may have arisen due to potential imbalanced difficulty at the two categories of questions (PRS vs. non‐PRS). Therefore, a word of caution is needed when comparing and contrasting PRS and non‐PRS contribution to the learning gain of the S‐group. Besides the suggested ‘inherited’ difference in questions complexity between PRS and non‐PRS, which is supported by hypothesis testing (Fig. S3), other parameters may have contributed to the improved performance at non‐PRS. For instance, most non‐PRS activities had a duration of 2 or 3 min as opposed to the 45 s for the PRS activities. So one hypothesis could be that the longer interaction with the concept via, that is pair discussion or the matching terms activity, may better support memorising and learning. Interestingly, a study which compared PRS vs. non‐PRS within the peer instruction model reports that clickers may seem at first instance motivating but no significant difference was detected in the conceptual learning gain, and therefore, peer instruction is viewed as an effective but clicker‐independent instructional approach [53]. Furthermore, a recent German study [54] reports positive students’ attitudes towards PRS, higher motivation and confidence as well as a request for PRS integration in more courses. However, their data could not confirm a long‐term positive effect on assessment results implying that PRS effect on actual learning gain could be less profound than its marketing profile and popularity of usage. In this context, it is worth mentioning that our results (Questions F12 and F13; Fig. 6B) suggest a preference towards non‐PRS activities with 79% of the S‐group students to strongly prefer non‐PRS activities over PRS. This of course does not mean that students did not enjoy the PRS activities. On the contrary, at free‐text comments of QUE2 (F16–F18; Fig. 7, Table S7) positive attitudes towards PRS have been noted.

Altogether, from our data we cannot attribute the enhanced outcome of the Sandwich Model on PRS incorporation. It seems that the Sandwich Model effect is PRS‐independent. However, future experimental trials with balanced non‐PRS vs. PRS activities are required in order to elucidate the PRS potential within the Sandwich Model. From a practical point of view, a mixture of PRS and non‐PRS activities may serve well the lecturer (i.e. time management) and the students (i.e. due to positive students’ attitudes) even if the learning gain is not arising directly from the PRS intervention but rather from the architecture of the Sandwich Model.

### The student experience with the Sandwich‐based epigenetic lecture: insights into student engagement and satisfaction

After evaluating the student learning outcome by a formative assignment (QUE1), we then examined students’ perceptions with regard to active learning strategies as presented at the form of the Sandwich Model with both PRS and non‐PRS components. This was based on the QUE2 feedback questionnaire (Appendix S3), which was comprised by three parts: (a) nine five‐point scale questions common for both the C‐group and the S‐group (F1–F9), (b) six additional five‐point scale questions addressed only at S‐group (F10–F15) and (c) three free‐text comment questions (F16–F18) addressed at both groups. Our student feedback analysis revealed valuable insights with regard to student perceptions of their learning experience with the Sandwich Model.

Firstly, comparison of the C‐group and the S‐group responses at the first part of QUE2 (F1–F9) revealed that the S‐group students had systematically significantly more positive attitudes in 5/9 questions (Fig. 6A). For instance, they were more confident about achieving the learning objectives (F1), found the content definitely more interesting (F3) and provided more rewarding comments for lecturer’s skills and performance (i.e. F6‐explaining difficult concepts, F7‐motivating others, F8‐encouraging participation). Since this is the first randomised trial about the Sandwich Model, these results demonstrate that this didactic approach has a powerful impact on student experience. One important element of this model is that it places emphasis on the structure. Evidence from existing literature suggests that structure is an important parameter of course design which positively affects performance in biology classes [55]. As the Carnegie Hall hypothesis states, a highly structured design with incorporation of active learning strategies reduces the achievement gap previously reported in students from disadvantaged backgrounds. In this context, and if one considers the lecture as the snapshot of a course, it is not surprising that the strict architecture of the Sandwich Model leads to highly significant student benefits as opposed to traditional lecturing.

Besides structure, the idea that active learning enhances learning gain is not new. Research has shown that active teaching strategies have important educational benefits [5, 8, 56]. However, it is reported that academics still favour traditional lecturing over active learning [4]. Interestingly, among reasons for lecturers’ resistance to incorporation of active learning strategies is the fear about unsatisfactory student evaluation [14, 57]. This fear is discussed in the study by Deslauriers *et al*., which describes an inherent student bias against active learning limiting its benefits. Of note, students in active learning classes as opposed to traditional lectures had the perception that they learned less, while in reality, they learned more [17]. In our study, this inherit bias was not detected in the comparisons of the C‐group vs. S‐group. The reason for that could be linked to evidence from the literature which state that lecturer’s prior promotion of activities is essential for positive students’ attitudes towards active learning strategies [16]. Indeed, as part of our experimental design, we informed the students well in advance via different methods such as blackboard announcements, participant information sheet online at module cell and circulated as hard copy in the lecture hall, promotion by the lecturer prior to the teaching session during previous lectures, as well as the promotion action from the year’s representatives. Although this is a plausible explanation, with our data we cannot test and quantify the impact of promoting activities in students’ perceptions for the Sandwich Model.

When we looked at the results of the second part of the feedback (F10–F15: questions that only the S‐group had answered) (Fig. 6B), we recognised an overall positive attitude which was characterised by evidence of high satisfaction (F10.I enjoyed…, F1..I found useful), clear preference for more interactive lectures in the future (F15) and a link between performance and activities in‐class. However, there was not possible to attribute satisfaction clearly to the incorporation of PRS activities; on the contrary, it seems that when students had to pick up PRS or non‐PRS activities as their favourite intervention, they voted mostly for the non‐PRS as indicated by a double question (F12, F13). Evidence from literature suggests a positive correlation between PRS incorporation and student satisfaction [58]; however, whether PRS are preferred or superior over other active learning interventions is not clear. The reason for the preference of the overall highly satisfied C‐group cohort towards non‐PRS activities over PRS activities is hard to be identified by our study data. One hypothesis could be that the non‐PRS activities allowed for a strong social component (i.e. discussion in‐pair, peer teaching) as well as a longer duration (2–3 min). PRS activities were very short (45 s) and individual.

By processing the free‐text commenting results and coding them to a five‐point scale (Appendix S3) (F16–F18), we showed that the S‐group participants did not only provide no negative comments, but they also felt significantly more engaged, demonstrated curiosity by asking relevant questions (i.e. biomarkers and their relation to cancer) and enjoyed the interactive nature of the lecture. On the contrary, C‐group participants were more concerned about content relevance for the examinations, considered the topic to be difficult and seemed relatively overwhelmed with the level of detail and pace. The last point may seem at first sight as a paradox since the group C had a less time‐intensive didactic approach (Fig. 3A) but on the same time it speaks for the didactic benefits of the Sandwich Model.

As far as student engagement is concerned, the free‐text comment analysis (Fig. 7) revealed significantly stronger positive perceptions upon application of the Sandwich Model for all three questions. Regarding student satisfaction, the C‐group participants were overall not critical or unsatisfied; however, they provided significantly fewer positive comments at 2/3 questions (F17, F18).

Overall, we found that the increased frequency of positive comments in the S‐group, compared with the C‐group, was statistically significant in the vast majority of the feedback questions (Figs 6 and 7). These results suggest that the Sandwich Model is a didactic principle with clear impact on student perceptions about own engagement, learning and satisfaction. Whether the positive attitudes emerging from the Sandwich Model application can be reflected to actual long‐term learning gain is an important research question that remains to be answered. As mentioned above, Deslauriers *et al*. have reported that active learning strategies lead often to lower levels of feeling of learning that the actual learning gain. This can be caused by (a) the cognitive fluency of lectures which can be misleading, (b) poor metacognition due to lack of prior subject knowledge and/or (c) lack of prior exposure to intense active learning [17]. So actually, students learn more but they feel that learned less which is at variance with what we have observed in the present study. Since in our experiment the negative association between feeling of learning and actual learning was not detected, it is therefore reasonable to ask: – firstly – why (which of the three above factors may have led to this) and – secondly – whether the feeling of learning due to the Sandwich Model application can be transformed to long‐term learning gain. From our study, we have strong indications that the positive attitudes towards the model are linked at least to short‐term learning gain as indicated by the formative assessment outcome. However, larger and across the academic year studies are required for linking the feeling of learning to actual long‐term learning gain for the Sandwich Model.

## Conclusion

The Sandwich Model can be seen as a potential powerful didactic tool which has not been broadly explored and in‐depth understood. Described mainly in Germany more than a decade ago, with significant amount of literature sources also in German, it has not received the scientific attention it may ‘deserve’. The Sandwich Principle is linked to active learning; however, its architecture is distinct and its power remains to be confirmed across disciplines and teaching forms (i.e. lectures, tutorials, practicals, remote teaching). Our study is the first randomised trial which evaluates in a controlled system the effect of the model on learning outcome and students’ perceptions. We provide compelling experimental evidence that the Sandwich Model supports learning in‐class and that students find the Sandwich‐based lectures more engaging, motivating and enjoyable. In conclusion, we have shown that the Sandwich Model enhances learning outcome and student satisfaction and that PRS may contribute to this success. An advantage of PRS vs. non‐PRS activities was not documented although students were favouring non‐PRS activities over PRS. Altogether, our study offers a rationale for applying the Sandwich Model in teaching epigenetics and other advanced biological and medical concepts.

## Conflict of interest

The authors declare no conflict of interest.

## Author contributions

GK supported the study design, processed and digitalised experimental data and wrote the paper. EK conceived and designed the study, delivered teaching, supervised activities, analysed data and wrote the paper.

## Supporting information


**Fig. S1**. Responses derived from Personal Response System (Turning Point) in‐class for S‐group (*n* = 54).
**Fig. S2.** Comparison of prior knowledge and skills between the two group by using end of first year results.
**Fig. S3**. Hypothesis testing for identifying the impact of PRS incorporation within the Sandwich Model.
**Fig. S4**. Feedback Questionnaire Results (QUE2) for F1–F9 for the whole study group.

**Table S1**. Multiple Choice Questionnaire (QUE1) results for control group (C‐group).
**Table S2**. Multiple Choice Questionnaire (QUE1) results for Sandwich‐ group (S‐group).
**Table S3**. Correspondence between active learning activity within the Sandwich‐model at QUE1 questions including information on time occurring within teaching session.
**Table S4**. Results from Personal Response System in‐class (Tuning Point) for S‐group (total 54 students): engagement, correct answers and comparison with QUE1 Results.
**Table S5**. Summary of Results from Feedback Questionnaire (QUE2).
**Table S6**. Descriptive Statistics: Summary of Results from Feedback Questionnaire (QUE2).
**Table S7**. Comments received on Questions F16–F18 of the Feedback Questionnaire (QUE2) from both C‐ and S‐group.

**Appendix S1**. PowerPoint presentation for the lecture delivered as part of the module BIOM2001 for the purpose of the current study.

**Appendix S2**. Questionnaire QUE1.

**Appendix S3**. Feedback Questionnaire (QUE2).

## Data Availability

The data that support the findings of this study are available in the supplementary material of this article (Figures S1–S4, Tables S1–S7, Appendix [Link], [Link], [Link]).
