# Supplementary material for: A Sandwich‐model experiment with personal response systems on epigenetics: insights into learning gain, student engagement and satisfaction
Source: FEBS Open Bio. 2021 Mar 29;11(5):1282–98. doi: 10.1002/2211-5463.13135 (PMC8091589; doi:10.1002/2211-5463.13135)
Supplement: Supplementary file 1 — Fig. S1. Responses derived from Personal Response System (Turning Point) in‐class for S‐group (n = 54). Fig. S2. Comparison of prior knowledge and skills between the two group by using end of first year results. Fig. S3. Hypothesis testing for identifying the impact of PRS incorporation within the Sandwich Model. Fig. S4. Feedback Questionnaire Results (QUE2) for F1–F9 for the whole study group. [file FEB4-11-1282-s005.docx]

**Supplemental Figure 1.** Responses derived from Personal Response System (Turning Point) in-class for S-group (n=54). PRS were employed six times and were linked to QUE1 assessment at the end of the class. Details on each question can be found on Supplemental Files A and B. NE=non-engaged. With green is highlighted the right answer, with red the wrong.

**Supplemental Figure 2.** Comparison of prior knowledge and skills between the two group by using end of first year results. Differences between C-group (M_C-group_=72.18, SD=11.94) vs. S-group (M_S-group_=68.81, SD=14.01) were not significant [ t(97)=1.271, p=0.2068,unpaired t-test].

**Supplemental Figure 3.** Hypothesis testing for identifying the impact of PRS incorporation within the Sandwich Model.  ****

**Supplemental Figure 4.** Feedback Questionnaire Results (QUE2) for F1-F9 for the whole study group (N=99 students, C-group and S-group together). Questions have been sorted based on the highest positive attitude scores. For instance, 99% of the student cohort agreed/strongly agreed that lecturer was well prepared and organised (F5) followed by 91% of the students who agreed/strongly agreed that lecturer explained new terms and difficult concepts clearly.
