# Supplementary material for: A Sandwich‐model experiment with personal response systems on epigenetics: insights into learning gain, student engagement and satisfaction
Source: FEBS Open Bio. 2021 Mar 29;11(5):1282–98. doi: 10.1002/2211-5463.13135 (PMC8091589; doi:10.1002/2211-5463.13135)
Supplement: Supplementary file 3 — Appendix S1. PowerPoint presentation for the lecture delivered as part of the module BIOM2001 for the purpose of the current study. [file FEB4-11-1282-s003.pdf]

Supplemental File A. Powerpoint presentation for the lecture delivered as part of the module BIOM2001 for the purpose of study. The PowerPoint version presented below includes popular and correct answers and it represents the version which was upload at Blackboard post-lecture. During delivering the lecture for the C-group the slides with the activities were hidden (indicated with grey bubbles) awhile during delivering the lecture for S-group PRS and non-PRS activities slides where included but not the correct answers.

Epigenetics & Epigenomics

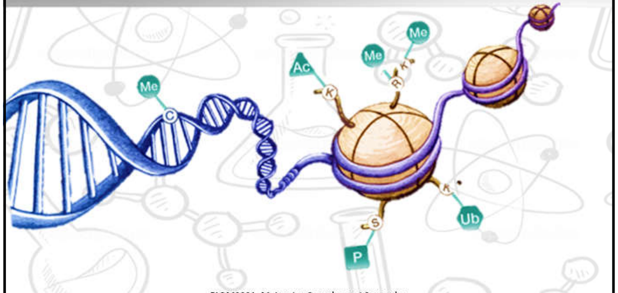

BIOM2001- Molecular Genetics and Genomics

Dr. Efterpi Kostareli

1

Learning Objectives

By the end of the lecture you will be able to …

PART 1: Epigenetic modifications and epigenomics

1. Provide the definition of epigenomics and epigenetics
2. Define nucleosome and describe its structure and function
3. Compare heterochromatin and euchromatin
4. Mention the epigenetic modifications of DNA and histone tails
5. Give the definition of DNA methylation, CpG island and mention by which enzymes DNA methylation is mediated

Learning Objectives

PART 2: The importance of the epigenetic modifications

1. Explain why tortoiseshell cats are only female
2. Describe the phenomenon of imprinting
3. Mention human diseases caused by aberrant epigenetic modifications
4. Mention available methods for epigenetic analysis

PART 3: DNA methylation & cancer

1. Describe the role of DNA methylation in cancer
2. Explain the concept of DNA methylation-based biomarkers and their applications in cancer.
3. Provide two examples of cancer-related DNA methylation biomarkers

Dr. Efterpi Kostareli 2

2

1

Epigenetic modifications & Epigenomics

PART 1: Learning goals

1. Provide the definition of epigenomics and epigenetics
2. Define nucleosome and describe its structure and function
3. Compare heterochromatin and euchromatin
4. Mention the epigenetic modifications of DNA and histone tails
5. Give the definition of DNA methylation, CpG island and mention by which enzymes DNA methylation is mediated

3

I. Epigenetic modifications and epigenomics | II. The importance of the epigenetic modifications | III. DNA methylation & Cancer

Epigenetics & Epigenomics: Definitions

The word "epigenetic" literally means "in addition to changes in genetic sequence."

Epigenetics:
is the study of **heritable changes** in cellular phenotype caused by mechanisms other than changes in the underlying DNA sequence

Epigenomics:
is the study of genome-wide changes in gene expression and cellular phenotype

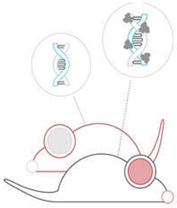

LO: 1.1 Dr. Efterpi Kostareli 4

Weinhold, 2006

4

I. Epigenetic modifications and epigenomics | II. The importance of the epigenetic modifications | III. DNA methylation & Cancer

Epigenetics & Epigenomics

Some examples of epigenetic changes in the phenotype:

- Stem cell differentiation
- Lyonisation -X chromosome inactivation. (see LO. 2.1)
- Imprinting –differential expression of genes inherited from the male and female parent. (see LO. 2.2)

LO: 1.1 Dr. Efterpi Kostareli 5

5

I. Epigenetic modifications and epigenomics | II. The importance of the epigenetic modifications | III. DNA methylation & Cancer

Epigenetics & Epigenomics

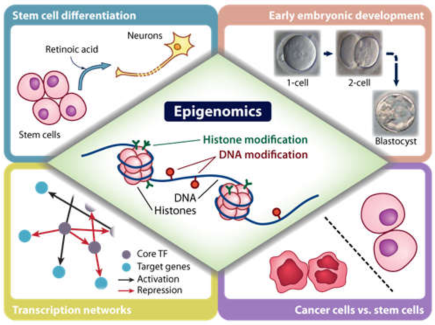

LO: 1.1 Dr. Efterpi Kostareli 6

6

So… Epigenetics…Whay it Matters?

Three Major Reasons:

i. Epigenetic mechanisms are involved in many fundamental areas of biology

ii. Epigenetic regulation plays a central role in gene expression, so is involved in disease processes.

iii. A large number of novel enzymes and protein complexes are involved in epigenetic processes, and are potential targets for small-molecule inhibition

7

Epigenetic changes are:

45

a. Heritable changes in cellular phenotype caused by mechanisms other than changes in the underlying DNA sequence.

b. Heritable changes in cellular phenotype caused by DNA sequence.

c. Heritable changes in cellular phenotype caused by DNA sequence and other mechanisms other than changes in the underlying DNA sequence.

d. Heritable and non-heritable changes in cellular phenotype by mechanisms caused by specific enzymes.

Hidden for C-lecture

8

Epigenetic changes are:

a. Heritable changes in cellular phenotype caused by mechanisms other than changes in the underlying DNA sequence.

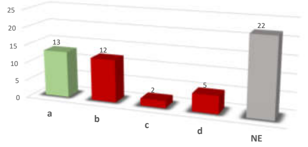

Only avaliable at Blackboard version

9

Chromatin Structure

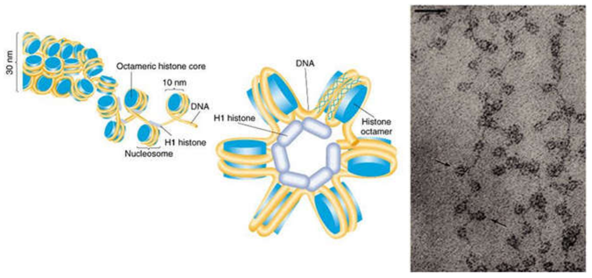

Olins, D. E. & Olins, A. L. Chromatin history: our view from the bridge. Nature Reviews Molecular Cell Biology 4, 811 (2003). © 2003 Nature Publishing Group

10

Chromatin Structure

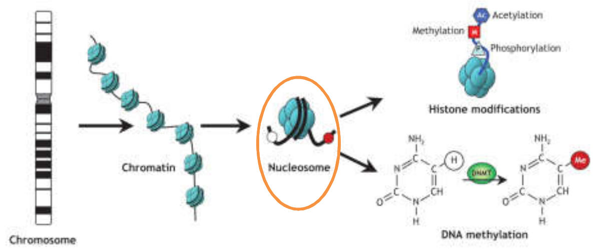

https://www.youtube.com/watch?v=mjIaGlhH0Ys

11

Structure of the nucleosome

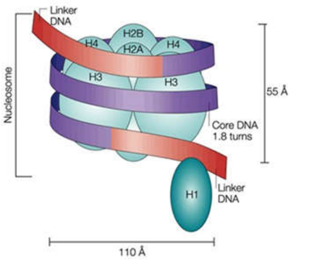

The nucleosome core particle contains:
2 copies of each **histone protein**
(H2A, H2B, H3 and H4)
146 basepairs (bp) of **superhelical DNA**
wrapped around this histone octamer

Nature Reviews Immunology 2, 162-174 (March 2002) | doi:10.1038/nri747

12

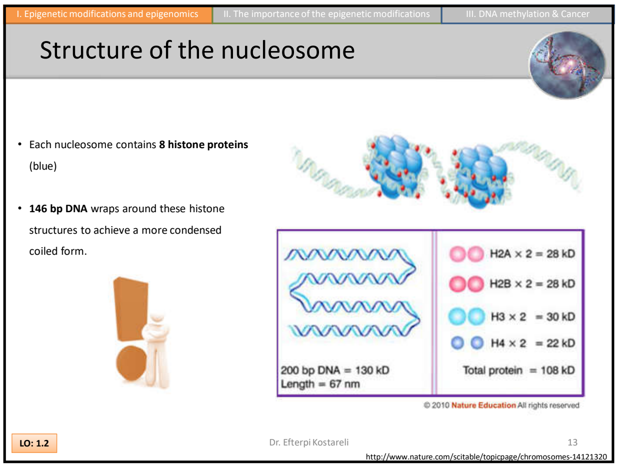

13

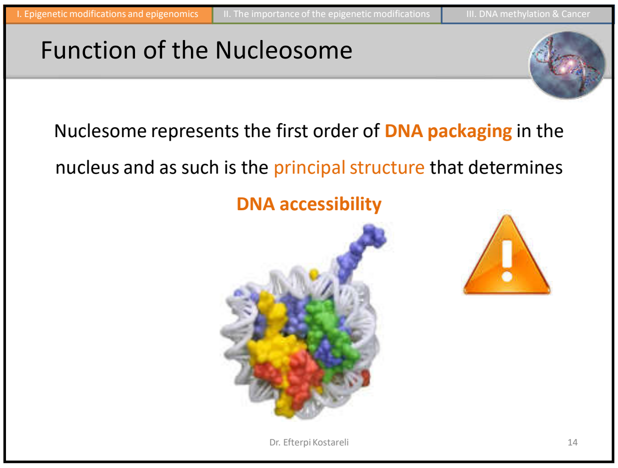

14

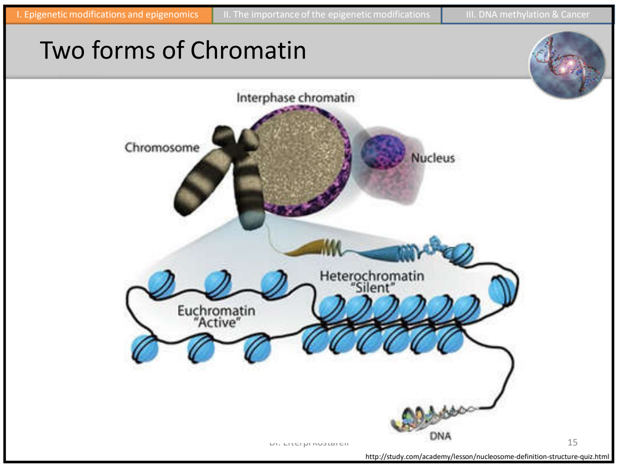

15

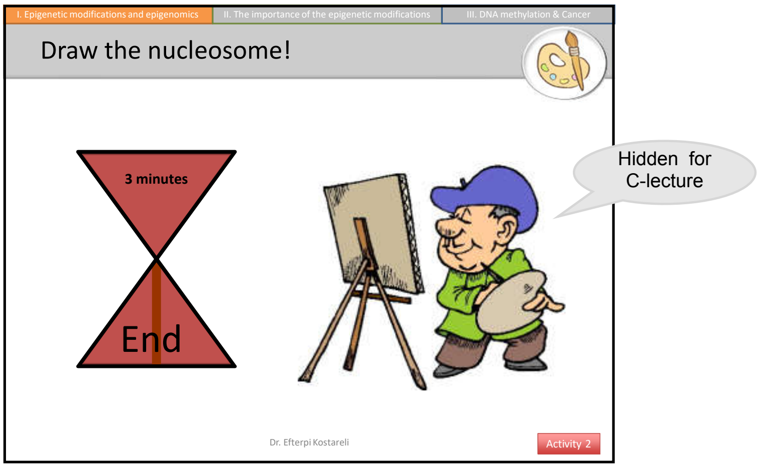

16

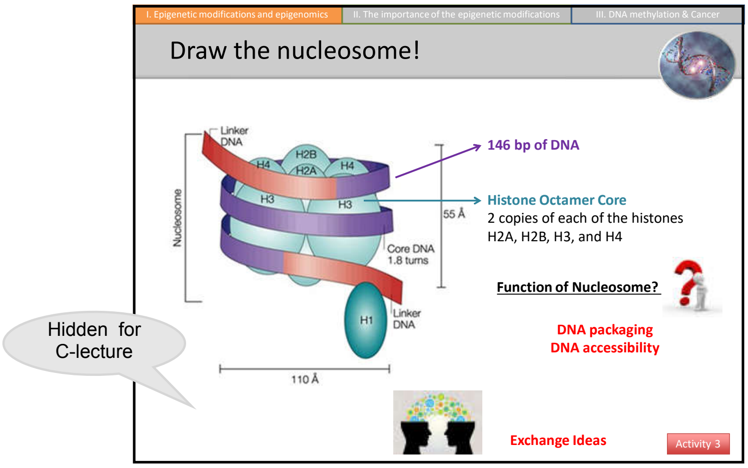

17

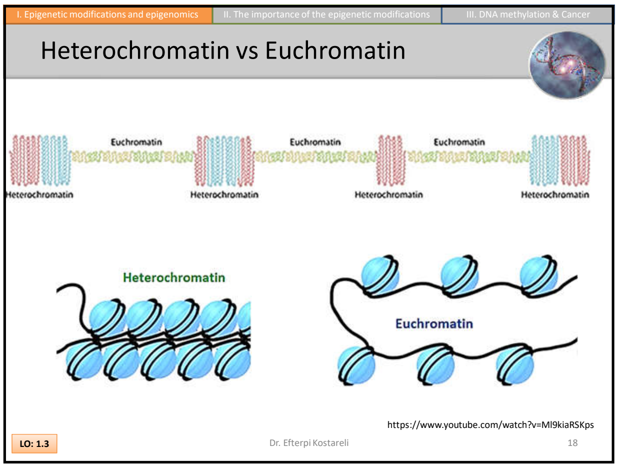

18

Heterochromatin vs Euchromatin

EU-CHROMATIN	HETERO-CHROMATIN
the light-colored bands	dark-colored bands
looser DNA packing-less condensed	tighter DNA packaging-highly condense
loosely coiled regions	compactly coiled regions
contains less DNA	contains more DNA
early replicative (throughout S-phase)	late replicative (in late S-phase)
eukaryotes, cells with nuclei, and prokaryotes, cells without nuclei	found only in eukaryotes
at chromosome arms	at centromeres and telomeres
contains unique sequences	contains repetitious sequences
gene-rich	gene-poor
recombination during meiosis	no meiotic recombination

https://www.youtube.com/watch?v=MI9kiaRSKps

LO: 1.3

19

Types of Epigenetic Modifications

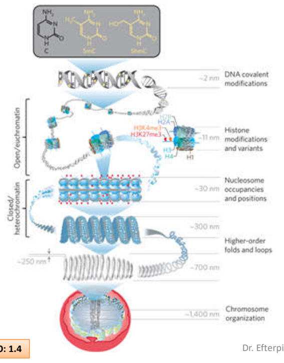

- **DNA modifications:**
  - DNA Methylation
  - DNA Hydroxymethylation
- **Histone modifications:**
  - Histone Methylation
  - Histone Acetylation
  - Histone Phosphorylation
  - Histone Ubiquitylation
  - Histone Sumoylation

Aguila & Craighead (2013)

LO: 1.4

20

Epigenetic modifications of histone tails

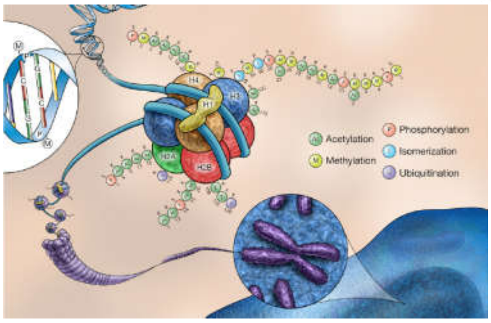

LO: 1.4

21

DNA methylation and CpG islands: definitions

- DNA methylation is an epigenetic mechanism that occurs by the addition of a methyl (CH3) group to DNA
- ''CpG islands" are clusters of CpGs with shared methylation status.
- Methylation of CpG islands at gene promoters prevents activation of enhancers (Gene Expression is Repressed)

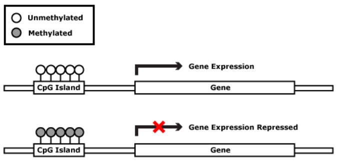

LO: 1.5

22

DNMTs: the enzymes of DNA methylation

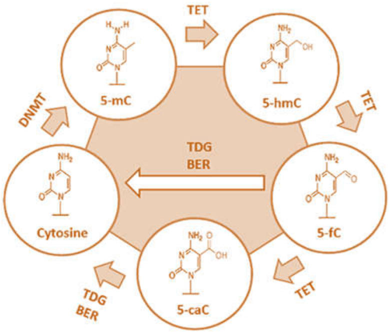

LO: 1.5

23

DNMTs: the enzymes of DNA methylation

The addition of methyl groups is controlled at several different levels in cells and is carried out by a family of enzymes called

DNA MethylTransferases (DNMTs)

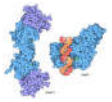

- Three DNMTs (DNMT1, DNMT3a and DNMT3b) are required for establishment and maintenance of DNA methylation patterns.
- Two additional enzymes (DNMT2 and DNMT3L) may also have more specialized but related functions.

LO: 1.5

24

DNMTs: the enzymes of DNA methylation

- DNMTs
  - De novo
    - DNMT3A
    - DNMT3B
    - → Methylation during embryonic developments
  - Maintenance
    - DNMT1
    - → Methylate hemi-methylated sites generated during DNA replication

LO: 1.5

25

I. Epigenetic modifications and epigenomics | II. The importance of the epigenetic modifications | III. DNA methylation & Cancer

Which of the following sentences is TRUE for EYchromatin

Quiz

45

a. Euchromatin is a lightly packed form of chromatin (DNA, RNA and protein) rich in genes.

b. Euchromatin is a tightly packed form of chromatin (DNA, RNA and protein).

c. Euchromatin is gene- poor

d. Euchromatin appears in dark-coloured bands when chromatin is stained

Hidden for C-lecture

Activity 4

26

I. Epigenetic modifications and epigenomics | II. The importance of the epigenetic modifications | III. DNA methylation & Cancer

Which of the following sentences is TRUE for EYchromatin

Quiz

a. Euchromatin is a lightly packed form of chromatin (DNA, RNA and protein) rich in genes.

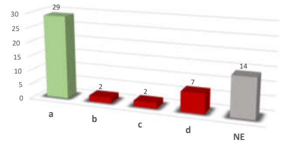

Only avaliable at Blackboard version

Activity 4

27

I. Epigenetic modifications and epigenomics | II. The importance of the epigenetic modifications | III. DNA methylation & Cancer

Which of the following sentences is TRUE for HETERO-chromatin

Quiz

45

a. Heterochromatin is loosely packed form of chromatin than euchromatin

b. Heterochromatin is present in both prokaryotes and eukaryotes while euchromatin is only present in eukaryotes.

c. Heterochromatin is easily and highly stained (dark-coloured bands).

d. Heterochromatin is highly active

Hidden for C-lecture

Activity 5

28

I. Epigenetic modifications and epigenomics | II. The importance of the epigenetic modifications | III. DNA methylation & Cancer

Which of the following sentences is TRUE for HETERO-chromatin

Quiz

c. Heterochromatin is easily and highly stained (dark-coloured bands).

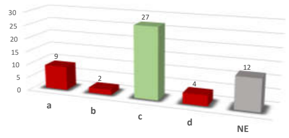

Only avaliable at Blackboard version

Activity 5

29

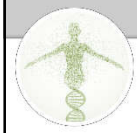

2

The importance of the epigenetic modifications

PART 2: Learning goals

1. Explain why tortoiseshell cats are only female
2. Describe the phenomenon of imprinting
3. Mention human diseases caused by aberrant epigenetic modifications
4. Mention available methods for epigenetic analysis

30

X-inactivation & Tortoiseshell cats

- X inactivation: in female animals the 2nd X chromosome condenses into a Barr body
- A different X will be randomly inactive in different cells.
- If a female is heterozygous for a particular gene located on the X chromosome she will be a mosaic in this character (tortoiseshell cats)

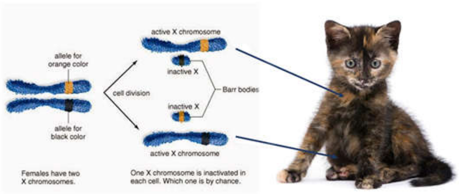

LO: 2.1

31

Tortoiseshell cats can be only female

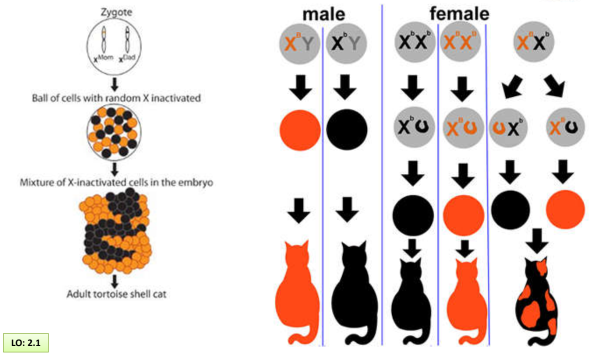

LO: 2.1

32

Discuss with your neighbour

Hidden for C-lecture

Why the Tortoiseshell cats can be only female?

What is the role of Epigenetics on that?

2:00

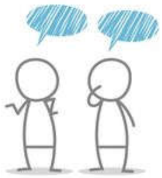

Dr. Efterpi Kostareli

Activity 6

33

Genomic imprinting

Genomic imprinting is the epigenetic phenomenon by which certain genes are expressed in a parent-of-origin-specific manner.

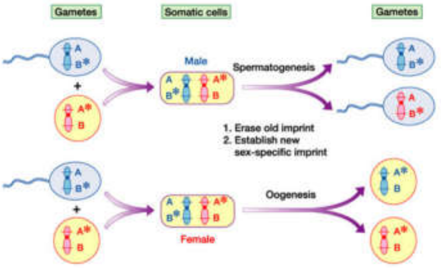

LO: 2.2

34

34

Genomic imprinting

Genomic imprinting is the epigenetic phenomenon by which certain genes are expressed in a parent-of-origin-specific manner.

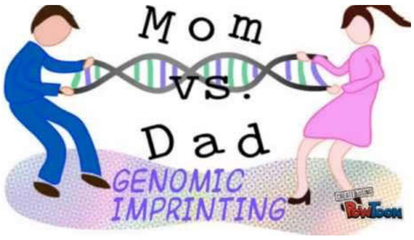

LO: 2.2

Dr. Efterpi Kostareli

35

35

Genomic Imprinting & Diseases

Deletions on chromosoome 15 can result in Prader-Willi or Angelman syndrome

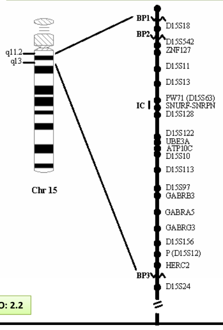

Prader-Willi Syndrome — Paternal
- -initial failure to thrive
- -distinctive facial features
- -developmenta delay
- -hypogonadism

Angelman Syndrome — Maternal
- -seizures
- -jerky, uncoordinated movements
- -unprovoked smiling/laughter
- -lack of speech
- -severe developmental delay

LO: 2.2

i Kostareli

36

36

Epigenetic & Diseases

Cancer
Autoimmune disorders
Mental diseases
Diabetes
Metabolic syndrome
Male infertility
Abnormal birth weight

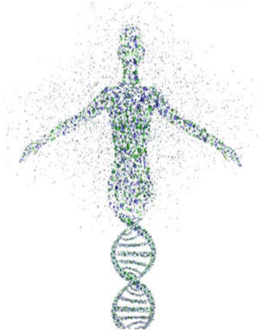

37

Epigenetic & Diseases

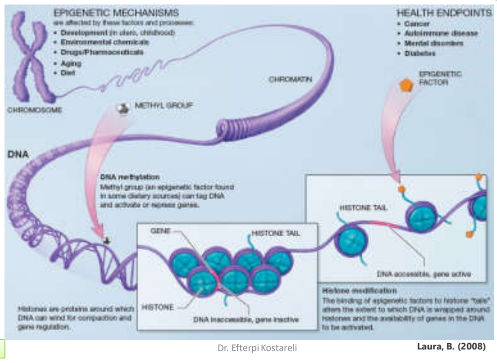

Laura, B. (2008)

38

DNA methylation and human disease

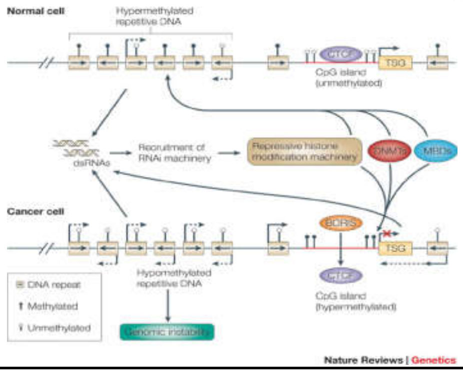

39

The enzymes that catalyse DNA methylation are called…

Hidden for C-lecture

- Write in a piece of paper the acronym and the detailed name of the DNA methylation enzymes
- Compare your result with your neighbours
- You have 1-minute-exchange the papers afterwards

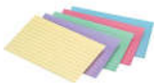

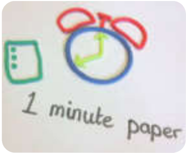

1:00

40

The enzymes that catalyse DNA methylation are called…

DNA MethylTransferases (DNMTs)

Only avaliable at Blackboard version

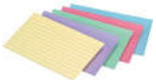

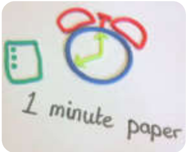

1:00

41

Epigenetics and Metabolic Syndrome

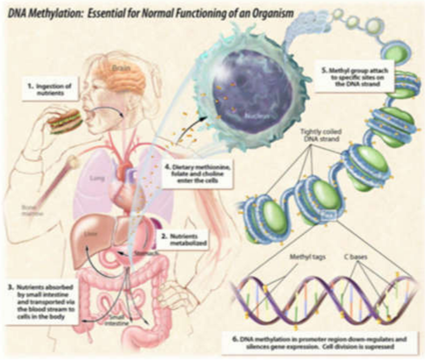

Amanda Mayer (2015)

42

Importance of Epigenetics & Technology

The importance of epigenetic modifications for health, development and disease is reflected on the intensified research for advanced technologies and novel methods

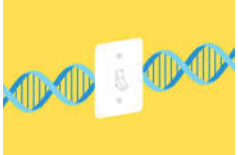

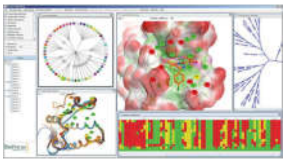

LO: 2.4

43

Epigenetic Analysis: methods

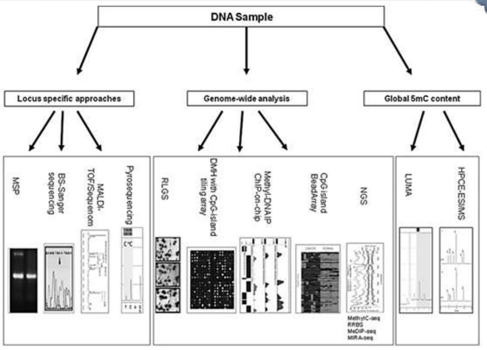

LO: 2.4

44

Epigenetic Analysis: methods

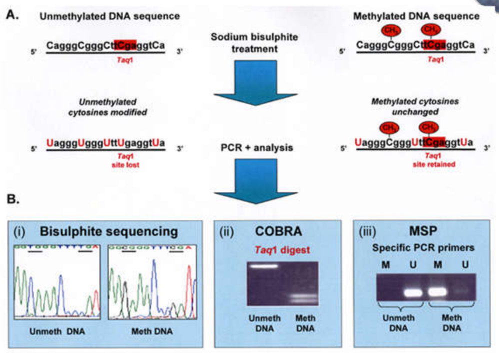

Source: Neurosurg Focus © 2005 American Association of Neurological Surgeons

LO: 2.4

45

Epigenetic Analysis: methods

Micro- and nanodevices for mapping DNA covalent modifications.

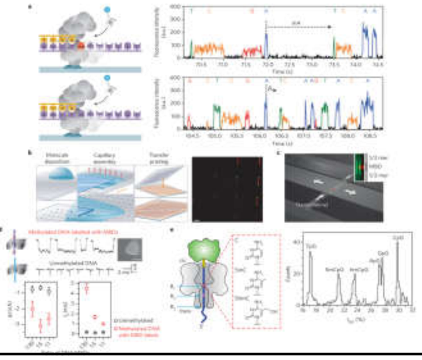

LO: 2.4

Aguila & Craighead (2013)

46

Which of the following techniques is used for DNA methylation analysis?

Quiz

a. Bisulphite Sequencing

b. Flow cytometry

c. Western blot

d. Crystallography

45

Hidden for C-lecture

Activity 8

47

Which of the following techniques is used for DNA methylation analysis?

Quiz

a. Bisulphite Sequencing

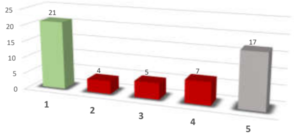

Only avaliable at Blackboard version

Activity 8

48

10 min Break

Please bring along:

The signed Consent Forms

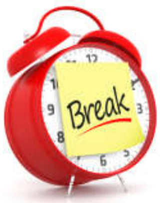

49

3

DNA methylation & Cancer

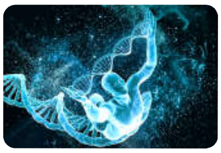

PART 3: Learning goals

1. Describe the role of DNA methylation in cancer
2. Explain the concept of DNA methylation-based biomarkers and their applications in cancer.
3. Provide two examples of cancer-related DNA methylation biomarkers

50

DNA methylation & Cancer

1. What is DNA methylation?
2. From which enzymes is mediated?
3. Where is DNA methylation located?
4. What is the role of DNA methylation in normal cells?

See LO: 1.5

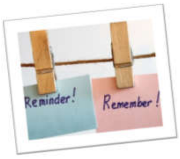

https://www.youtube.com/watch?v=W-S84J4zK9E
https://www.youtube.com/watch?v=EyWXbNqaCo4

51

DNA methylation & Cancer

1.What is DNA methylation?

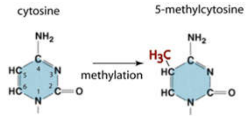

- DNA methylation is a type of chemical modification of DNA which involves the addition of a methyl group to the number 5 carbon of the cytosine, to convert cytosine to 5-methylcytosine.
- The most well characterized epigenetic mechanism.
- In humans, DNA methylation occurs in cytosines that precede guanines (dinucleotide CpGs) (The CpG notation is used to distinguish a cytosine followed by guanine from a cytosine base paired to a guanine).

52

DNA methylation & Cancer

2. From which enzymes is mediated?

The addition of methyl groups is controlled at several different levels in cells and is carried out by a family of enzymes called

DNA MethylTransferases (DNMTs)

- In humans, DNA is methylated by three enzymes, DNA methyltransferase 1, 3a, and 3b (DNMT1, DNMT3a, DNMT3b).
- DNMT3a and 3b are the de novo methyltransferases that set up DNA methylation patterns early in development.
- DNMT1 is the maintenance methyltransferase that is responsible for copying DNA methylation patterns to the daughter strands during DNA replication

53

DNA methylation & Cancer

3. Where is DNA methylation located?

- CpG sites are not randomly distributed in the genome - the frequency of CpG sites in human genomes is 1%, which is less than the expected (~4-6%).
- Around 60-90% of CpGs are methylated in mammals. DNA methylation frequently occurs in repeated sequences, and may help to suppress junk DNA and prevent chromosomal instability.
- There are regions of the DNA that have a higher concentration of CpG sites (> 60%), named the **CpG islands**, which tend to be located in the promoter regions of many genes.
- The CpG islands are usually not methylated.

54

DNA methylation & Cancer

4.What is the role of DNA methylation in normal cells?

Functions of DNA methylation

- Transcriptional gene silencing
- Maintain genome stability
- Embryonic development
- Genomic imprinting
- X chromosome inactivation (females)

55

CpG islands…

Quiz

a. are comprised of ACCTCTCTT motifs

b. are regions of the DNA with very low concentration of CpG sites (<20%)

c. exist only in human genome

d. tend to be located in the promoter regions of many genes

Hidden for C-lecture

56

CpG islands…

Quiz

d. tend to be located in the promoter regions of many genes

Only avaliable at Blackboard version

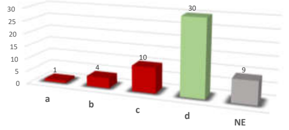

57

DNA methylation & Cancer

Hypomethylation – decrease methylation levels

- A lower level of DNA methylation in tumors as compared to their normal-tissue counterparts was one of the first epigenetic alterations to be found in human cancer.
- Global hypomethylation of DNA sequences that are normally heavily methylated may result in
  - **Chromosomal instability**
  - **Increased transcription from transposable elements**
  - **An elevated mutation rate due to mitotic recombination**
- Promoter region CpG islands demethylation of proto-oncogenes will activate the repressed gene expression

58

DNA methylation & Cancer

Hypermethylation – increase methylation levels

- **Hypermethylation of the CpG islands in the promoter** regions of tumor-suppressor genes is a major event in the origin of many cancers.
- **Hypermethylation of the CpG island promoter** can inactivate tumor-suppressor genes, affect genes involved in the cell cycle, DNA repair, and the metabolism of carcinogens, all of which are involved in the development of cancer.
- The profiles of hypermethylation of the CpG islands in tumor-suppressor genes are specific to the cancer type.

59

DNA methylation & Cancer

1. **Aberrant DNA methylation pattern in cancer cells**
2. **Global DNA hypomethylation of the genome**
3. **Localized DNA hypermethylation**

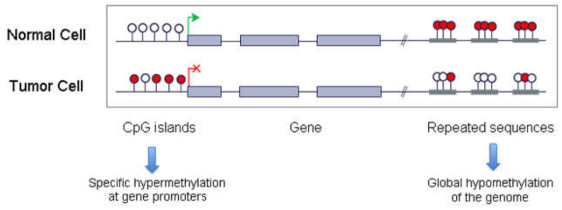

http://atlasgeneticsoncology.org/Deep/DNAMethylationID20127.html

60

The most typical example: Promoter Hypermethylation

A Simplified view

normal cell:

cancer cell:

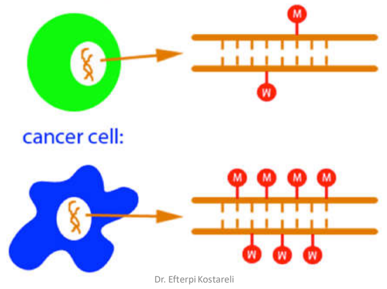

LO: 3.1

61

DNA methylation patterns in normal vs cancer cells

LO: 3.1

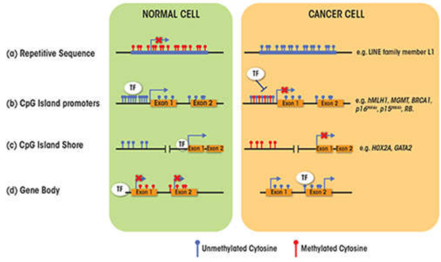

(a) Repetitive sequences generally are methylated at cytosine nucleotides in normal cells. Global loss of methylation in cancer cells leads to chromosomal instability and activation of endoparasitic sequences.
(b) CpG islands in promoter sequences typically are unmethylated in normal cells whereas they can become hypermethylated in cancer cells, leading to transcriptional repression. Examples of genes affected are shown on the right. (
(c) Similar patterns are seen in CpG island shores, located in front (i.e., upstream) of promoters.
(d) CpGs located in gene bodies frequently are methylated in normal cells; this pattern is reversed in cancer cells, leading to initiation of transcription at several incorrect sites. [illegible]

62

DNA methylation changes leading to cancer

Altered DNA methylation in somatic cell (promoter region)
↓
Silence Tumor suppressor genes
↓
Promote tumoriogenic behaviour
- ↑ Cell proliferation
- Escape apoptosis
- Enhance invasivceness
↓
Increases the rate of mutations
↓
Gene instability
↓↓
CANCER

Figure 4: Flow chart of DNA methylation changes leading to cancer.

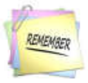

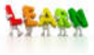

LO: 3.1

63

Teaching your Peers

Name mechanisms by which DNA methylation changes can lead to cancer

3:00

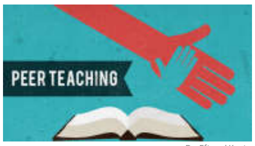

Hidden for C-lecture

Activity 10

64

DNA methylation & Cancer

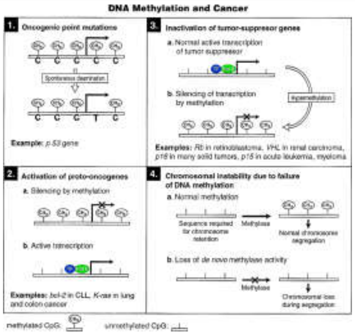

Models for the different mechanisms through which cytosine methylation can promote oncogenesis.

65

Genes altered by DNA methylation in cancer

Inactivation

- Rb (tumor supressor)
- Mlh1 (mismatch repair)
- VHL (oxidative stress response)
- $p16^{INK4A}$ (CDK inhibitor)

Activation

- parasitic sequences eg: retrotransposons
- activate homologous recombination?

LO: 3.1

66

Biomarkers: definition and discovery

Biomarker=a characteristic that is objectively measured and evaluated as an indicator of normal biological processes, pathogenic processes or pharmacologic responses to a therapeutic intervetion

Discovery → Confirmation → Validation & Refinement → Adoption

Identification ⟶ Established relevance to population ⟶ Identify clinical utility

LO: 3.2 Dr. Efterpi Kostareli 67

67

I. Epigenetic modifications and epigenomics | II. The importance of the epigenetic modifications | III. DNA methylation & Cancer

Biomarkers: types

Types of Biomarkers

**Prognostic biomarker** prvides information about patient outcome, regardless of therapy	**Predictive biomarker** used in advance of therapy to estimate the response to a specific treatment
**Pharmacodynamic** changes after treatment associated with target modulation by an agent	**Surrogate marker** marker to substitute for a clinical endpoint

LO: 3.2 Dr. Efterpi Kostareli 68

68

I. Epigenetic modifications and epigenomics | II. The importance of the epigenetic modifications | III. DNA methylation & Cancer

DNA methylation: applications & biomarkers

- EARLY DIAGNOSIS
  - Detection of CpG-island hypermethylation in biological fluids and serum
- PROGNOSIS
  - Hypemethylation of specific genes
  - Whole DNA methylation profiles
- PREDICTION
  - CpG island hypermethylation as a marker of response to chemotherapy
- PREVENTION
  - Developing DNMTs inhibitors as chemopreventive drugs to reactive silenced genes

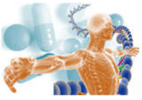

LO: 3.2 Dr. Efterpi Kostareli 69

69

I. Epigenetic modifications and epigenomics | II. The importance of the epigenetic modifications | III. DNA methylation & Cancer

DNA methylation biomarkers

- **A biological marker (biomarker)** is a biological molecule found in blood, other body fluids, or tissues that is an objective indicator of normal or abnormal process, or of a condition or disease
- **Cancer biomarkers signal the presence of cancer.** A cancer biomarker might be either a molecule secreted by the tumor itself, or it could be a specific response of the body to the presence of cancer. A biomarker can exist as related to DNA, RNA, micro-RNA, epigenetic changes, protein and even antibody expression.
- **Cancer-specific DNA methylation patterns** can be found in detached tumor cells in body fluids and biopsies, and they can be detected in free floating DNA that is released from dead cancer cells. Analysis of DNA methylation test results from cancer patients could facilitate the development of accurate biomarkers for early detection and diagnosis, prognosis and clinical decision support

Sommers et al., 2013

Dr. Efterpi Kostareli 70

70

I. Epigenetic modifications and epigenomics | II. The importance of the epigenetic modifications | III. DNA methylation & Cancer

DNA methylation biomarkers

Tissues that can be used to investigate DNA methylation biomarkers.

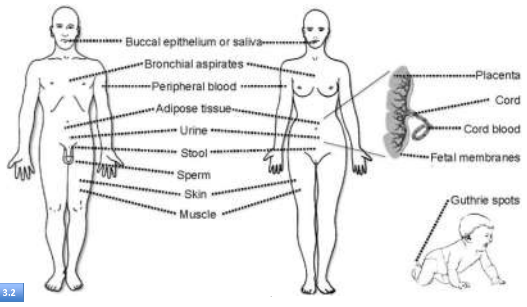

LO: 3.2 71

71

I. Epigenetic modifications and epigenomics | II. The importance of the epigenetic modifications | III. DNA methylation & Cancer

A prognostic biomarker:

Quiz

a. is a marker to substitute for a clinical endpoint

b. used in advance of therapy to estimate response to treatment

c. provides information about patient outcome, regardless of therapy

d. changes after treatment associated with target modulation by an agent

Hidden for C-lecture

Dr. Efterpi Kostareli Activity 11

72

A prognostic biomarker:

Quiz

c. provides information about patient outcome, regardless of therapy

Only avaliable at Blackboard version

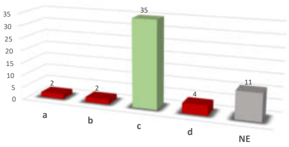

Activity 11

73

DNA methylation biomarkers

Commercially-available DNA methylation test kits for cancer.

Gene(s)	Type of Biomarker	Cancer	Diagnostic Test Kit
VIM	diagnostic	Colorectal	Cologuard (Exact Sciences)
SEPT9	diagnostic	Colorectal	Epi proColon (Epigenomics)<br>ColoVantage (Quest Diagnostics)<br>RealTime mS9 (Abbott)
SHOX2	diagnostic	Lung	Epi prolong (Epigenomics)
GSTP1<br>*APC*<br>*RASSF1A*	diagnostic	Prostate	ConfirmMDx (MDx Health)
MGMT	predictive	Glioblastoma	PredictMDx Glioblastoma (MDx Health)<br>SALSA MS-MLPA probemix ME011<br>Mismatch Repair genes (MRC-Holland)<br>PyroMark MGMT Kit (Qiagen)

LO: 3.2

74

Vimentin methylation in colorectal cancer

Colorectal Cancer: Cologuard test

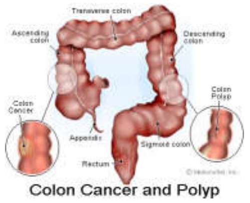

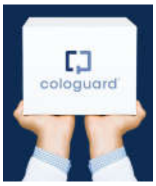

http://www.exactsciences.com/our-products/screening-test

LO: 3.3

75

GSTP1 gene in prostate cancer

normal prostate epithelium — 0/48=0%

basal cells+ columnar cells- → proliferative inflammatory atrophy — 4/64=6%

basal cells+ columnar cells+ → prostatic intraepithelial neoplasia — 22/32=69%

basal cells+ columnar cells- → localized prostate cancer — 30/33=91%

carcinoma cells- → metastatic prostate cancer

carcinoma cells-

GSTP1 CpG island hypermethylation

GSTP1 expression

LO: 3.3

76

GSTP1 gene in prostate cancer

ConfirmMDx (MDx Health)

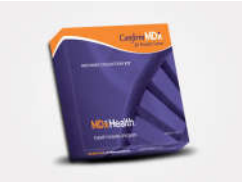

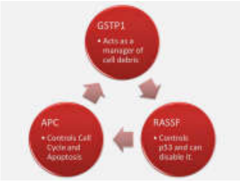

LO: 3.3

77

MGMT methylation in glioblastoma

- O6-DNA methylguanine methyl-transferase (MGMT) is a DNA repair enzyme that causes resistance to temozolomide, the current standard of care in the treatment of GBM.
- High expression of the MGMT (unmelthylated) enzyme is strongly correlated with poor patient outcomes.

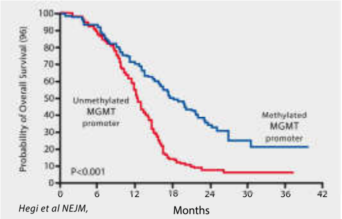

Hegi et al NEJM, 2005

LO: 3.3

78

Match the DNA methylation biomarker with the cancer type

1. Colorectal
2. Lung
3. Prostate
4. Glioblastoma

a. GSTP1
b. VIM
c. MGMT
d. SHOX2

Hidden for C-lecture

2:00

Work in Pairs

Activity 12

79

Match the DNA methylation biomarker with the cancer type

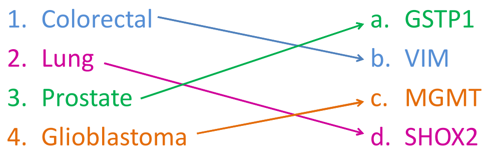

Only avaliable at Blackboard version

Work in Pairs

Activity 12

80

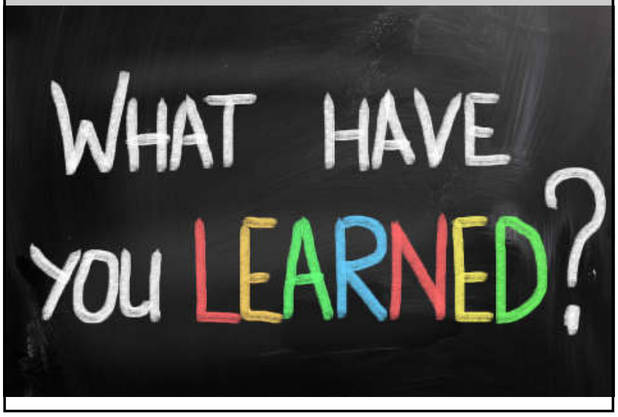

81

Learning Objectives

Learning Objectives

PART 1: Epigenetic modifications and epigenomics
1. Provide the definition of epigenomics and epigenetics
2. Define nucleosome and describe its structure and function
3. Compare heterochromatin and euchromatin
4. Mention the epigenetic modifications of DNA and histone tails
5. Give the definition of DNA methylation, CpG island and mention by which enzymes DNA methylation is mediated

PART 2: The importance of the epigenetic modifications
1. Explain why tortoiseshell cats are only female
2. Describe the phenomenon of imprinting
3. Mention human diseases caused by aberrant epigenetic modifications
4. Mention available methods for epigenetic analysis

PART 3: DNA methylation & cancer
1. Describe the role of DNA methylation in cancer
2. Explain the concept of DNA methylation-based biomarkers and their applications in cancer.
3. Provide two examples of cancer-related DNA methylation biomarkers

82

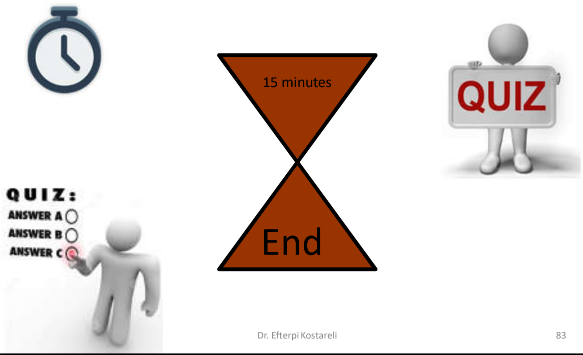

83
