# Supplementary material for: A Sandwich‐model experiment with personal response systems on epigenetics: insights into learning gain, student engagement and satisfaction
Source: FEBS Open Bio. 2021 Mar 29;11(5):1282–98. doi: 10.1002/2211-5463.13135 (PMC8091589; doi:10.1002/2211-5463.13135)
Supplement: Supplementary file 4 — Appendix S2. Questionnaire QUE1. [file FEB4-11-1282-s002.pdf]

QUESTIONNAIRE

Multiple choice questions- circle ONLY one of the 4 choices (ONLY one is CORRECT)

1. **Epigenetic changes are:**
   a. Heritable changes in cellular phenotype caused by mechanisms other than changes in the underlying DNA sequence.
   b. Heritable changes in cellular phenotype caused by DNA sequence.
   c. Heritable changes in cellular phenotype caused by DNA sequence and other mechanisms other than changes in the underlying DNA sequence.
   d. Heritable and non-heritable changes in cellular phenotype by mechanisms caused by specific enzymes.

2. **A nucleosome consists of:**
   a. 400 bp of DNA and 2 copies of the histones H2A, H2B, H3, and H4.
   b. 400 bp of DNA and 3 copies of the histones H2A, H2B, H3, and H4.
   c. 146 bp of DNA and 2 copies of each of the histones H2A, H2B, H3, and H4.
   d. 350 bp of DNA and 12 copies of the histones H2A, H2B, H3, and H4.

3. **The main function(s) of nucleosome is:**
   a. DNA packaging and determination of DNA accessibility
   b. Control of the end of DNA replication
   c. Protection of histone modifications
   d. Facilitation of RNA synthesis

4. **Which of the following sentences is TRUE for Euchromatin:**
   a. Euchromatin is a lightly packed form of chromatin (DNA, RNA and protein) rich in genes.
   b. Euchromatin is a tightly packed form of chromatin (DNA, RNA and protein).
   c. Euchromatin is gene- poor
   d. Euchromatin appears in dark-coloured bands when chromatin is stained

5. **Which of the following sentences is TRUE for Heterochromatin:**
   a. Heterochromatin is loosely packed form of chromatin than euchromatin
   b. Heterochromatin is present in both prokaryotes and eukaryotes while euchromatin is only present in eukaryotes.
   c. Heterochromatin is easily and highly stained (dark-coloured bands).
   d. Heterochromatin is highly active

6. **The tortoiseshell cats are:**
   a. Male or female
   b. Always male
   c. Always female
   d. Hermaphrodite

7. The enzymes that catalyse DNA methylation are called:

a. DNA methylacetylases (DNMAs)
b. DNA methyltransferases (DNMTs)
c. DNA methyltelomerases (DMTLs)
d. DNA novel-methylators (DNMTs)

8. Which of the following techniques is used for DNA methylation analysis?

a. Bisulphite Sequencing
b. Flow cytometry
c. Western blot
d. Crystallography

9. CpG islands:

a. are comprised of ACCTCTCTT motifs
b. are regions of the DNA with very low concentration of CpG sites (<20%)
c. exist only in human genome
d. tend to be located in the promoter regions

10. DNA methylation changes can lead to cancer by

a. silencing of tumor-suppressor genes
b. silencing of proto-oncogenes
c. increasing DNA stability
d. reducing cell proliferation

11. A prognostic biomarker:

a. is a marker to substitute for a clinical endpoint
b. used in advance of therapy to estimate response to treatment
c. provides information about patient outcome, regardless of therapy
d. changes after treatment associated with target modulation by an agent.

12. A commercially-available DNA methylation kit for glioblastoma test methylation levels at the following gene:

a. GATA4 (GATA Binding Protein 4)
b. MGMT (O6-DNA methylguanine methyl-transferase)
c. POLB(DNA polymerase beta)
d. SEPT9 (Septin 9)

Thank you for your participation!
