# Supplementary material for: A Sandwich‐model experiment with personal response systems on epigenetics: insights into learning gain, student engagement and satisfaction
Source: FEBS Open Bio. 2021 Mar 29;11(5):1282–98. doi: 10.1002/2211-5463.13135 (PMC8091589; doi:10.1002/2211-5463.13135)
Supplement: Supplementary file 5 — Appendix S3. Feedback Questionnaire (QUE2). [file FEB4-11-1282-s004.pdf]

TEACHING EVALUATION FORM

Topic: **Epigenetics & Epigenomics Lecture** Lecturer: **Dr Effie Kostareli**

Would you take a minute to answer the questions below and kindly provide your feedback on last week session?

I attended: morning session ☐ ***afternoon session*** ☐

Please if you attended the morning session do not reply to questions 10-15 (in blue)

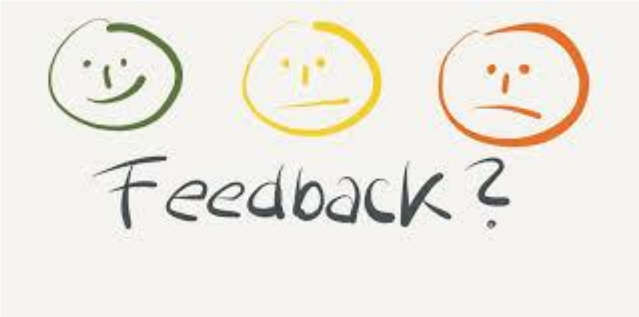

		I strongly agree	I agree	I neither agree/nor I disagree	I disagree	I disagree strongly	
1. The learning objectives of the lecture series were achieved	Strongly agree	☐	☐	☐	☐	☐	Disagree strongly
2.Material was presented in a logical manner	Strongly agree	☐	☐	☐	☐	☐	Disagree strongly
3. Overall, the content of lectures/workshop was interesting	Strongly agree	☐	☐	☐	☐	☐	Disagree strongly
4. Overall, I found the topic epigenetics challenging	Strongly agree	☐	☐	☐	☐	☐	Disagree strongly
5. The lecturer was well prepared and well organised	Strongly agree	☐	☐	☐	☐	☐	Disagree strongly
6. The lecturer explained new explained new terms and difficult concepts clearly	Strongly agree	☐	☐	☐	☐	☐	Disagree strongly
7. The lecturer motivated me to do my best work	Strongly agree	☐	☐	☐	☐	☐	Disagree strongly
8. The lecturer encouraged participation from students	Strongly agree	☐	☐	☐	☐	☐	Disagree strongly
9. Overall, I am satisfied by the quality of teaching delivered by the lecturer	Strongly agree	☐	☐	☐	☐	☐	Disagree strongly
10. I enjoyed interactive activities	Strongly agree	☐	☐	☐	☐	☐	Disagree strongly
11. I found interactive activities useful	Strongly agree	☐	☐	☐	☐	☐	Disagree strongly
12. I liked mostly the use of clickers (personal response systems)	Strongly agree	☐	☐	☐	☐	☐	Disagree strongly
13. I liked mostly other interactive activities (without clickers)	Strongly agree	☐	☐	☐	☐	☐	Disagree strongly
14. I performed well in MCQ test because of interactive activities in-class	Strongly agree	☐	☐	☐	☐	☐	Disagree strongly
15. I want more interactive lectures in the future	Strongly agree	☐	☐	☐	☐	☐	Disagree strongly

***Questions 10-15 were not requested for the feedback session of control group.**

16. What question(s) remain uppermost in your mind?

17. What did you enjoy/dislike most in the teaching session?

18. Any other comments

(Please use the space below to expand on any of your comments, or to add any other observations you think would be useful)

Thank you for your participation!
